# Screening and identification of key genes regulating fall dormancy in alfalfa leaves

**DOI:** 10.1371/journal.pone.0188964

**Published:** 2017-12-06

**Authors:** Hongqi Du, Yinghua Shi, Defeng Li, Wenna Fan, Guoqiang Wang, Chengzhang Wang

**Affiliations:** 1 College of Animal Science and Veterinary Medicine, Henan Agricultural University, Henan Key Laboratory of Innovation and Utilization of Grassland Resources, Zhengzhou, Henan, China; 2 School of Animal Science and Technology, Henan University of Science and Technology, Luoyang, Henan, China; NARO Institute of Fruit Tree Science, JAPAN

## Abstract

Fall dormancy (FD) determines the adaptation of an alfalfa variety and affects alfalfa production and quality. However, the molecular mechanism underlying FD remains poorly understood. Here, 44 genes regulating FD were identified by comparison of the transcriptomes from leaves of Maverick (fall-dormant alfalfa) and CUF101(non-fall-dormant), during FD and non-FD and were classified them depending on their function. The transcription of IAA-amino acid hydrolase ILR1-like 1, abscisic acid receptor PYL8, and monogalactosyldiacylglycerol synthase-3 in Maverick leaves was regulated by daylength and temperature, and the transcription of the abscisic acid receptor PYL8 was mainly affected by daylength. The changes in the expression of these genes and the abundance of their messenger RNA (mRNA) in Maverick leaves differed from those in CUF101 leaves, as evidenced by the correlation analysis of their mRNA abundance profiles obtained from April to October. The present findings suggested that these genes are involved in regulating FD in alfalfa.

## Introduction

Fall dormancy(FD) is defined as an adaptive growth characteristic of alfalfa in autumn in response to the reduction of daylength and drop in temperature. Alfalfa varieties are classified into three types, fall-dormant varieties (FD I-III), semi-dormant varieties(FD IV-VI), and non-dormant varieties(FD IIV-IX), according to plant regrowth after mowing in the autumn[1–3]. FD, which is one of the most important factors that influences plant adaptation, has a dramatic impact on the production of alfalfa[2].Therefore, FD is considered the primary evaluation index of alfalfa varieties in North America [4]. In this region, fall-dormant alfalfa is adapted to the cold climate, grows slowly after cutting in autumn, and has low yield, but exhibits strong cold-hardiness and overwintering ability, which non-dormant alfalfa does not possess[5]. The FD of fall-dormant alfalfa is stronger than that of semi-dormant and non-dormant alfalfa, and that of non-dormant alfalfa is the weakest [6–8]. Recently, it has been reported that environmental factors, such as photoperiod and temperature, regulate FD[9, 10]. Furthermore, photoperiod is considered a limiting FD-inducing factor, and complex interactions associated with daylength and temperature are known to occur[6].

Deeper insights into the molecular mechanisms underlying FD have been recently reported. Douglas identified genomic regions that control the FD of alfalfa, although the specific nucleotide sequences of these genomic regions remain unknown[11]. Wang et al. found that PHYA/PHYB mRNA content increases with the shortening of daylength and reduction in temperature[8]. Fan et al. identified 583 dormancy-related microRNAs via high-throughput sequencing, of which 28 microRNAs were found to play key roles in regulating dormancy[12]. Zhang et al. detected 2,064 differentially expressed genes in alfalfa leaves between dormant and non-dormant periods by transcriptome sequencing[13]. In addition, the reduction of indoleacetic acid (IAA), zeatin riboside (ZR), and gibberellins (GA3) in leaves of fall-dormant varieties is more significant than that of semi-dormant and non-dormant varieties. Further, increased levels of abscisic acid (ABA) were observed in semi-dormant and non-dormant varieties[14]. However, to the best of our knowledge, the genes, specifically those related to photoperiod and temperature, that regulate FD in alfalfa remain unknown.

In the present study, the candidate genes that regulate alfalfa FD were identified by comparing the leaf transcriptomes of Maverick (fall-dormant alfalfa) and CUF101 (non-fall-dormant alfalfa) at FD and non-dormant periods, the results of the bioinformatics analysis, and the data for gene function from previous studies. Finally, three genes regulating fall dormancy were identified by analyzing the changes in mRNA abundance of key candidate genes from April to October and by performing correlation analysis between their mRNA abundance and daylength and temperature. The present study establishes the foundation for highly targeted study of molecular mechanisms underlying FD.

## Materials and methods

### Plant materials and growth condition

Seeds of Maverick (FD I) and CUF101 (FD IX) of alfalfa standard varieties were introduced from the United States and planted in sandy loam soil at the Experimental Station of Henan Agricultural University (34°19 N, 113°35 E) by hand on October 1,2009, with 0.6m spacing between rows. The plants were irrigated regularly during drought, but they were not fertilized during growth. Weeds were controlled by hand or hoeing, and insects were controlled as required by hand. Leaf samples of the fall-dormant alfalfa variety collected in May and September are labeled DM and DS, respectively, and the samples of the non-dormant alfalfa variety collected in September are labeled NDS thereafter[13].

The plants were cut, then let grow for 14 days, the leaf samples were collected, which was done once a month. Three replicates of mature fresh leaves of both varieties were collected from the top of the plant between 08:00 and 09:00 on April 12, May 10, June 12, July 8, August 6, September 2, and October 18 in2011,and on April15, May11, June13, July9, August7, September5, and October16 in 2016.The samples were immediately frozen in liquid nitrogen, and stored at -80°C until use for RNA extraction and assessment of mRNA abundance of genes. Meanwhile, height of 30 randomly chosen plants was recorded to calculate the mean height for each plot. Leaf area was calculated based on the length and width of ten randomly chosen leaves of each plant [15]. Day length and temperature of each sampling day were also recorded.

Maverick and CUF101 grown under artificial growth conditions in a green house were exposed to different illumination times or different temperature. One group of plants including three plants of each variety was cultured at 24°C and a daily illumination (3000lux) of 8h, 12h, or 16h. Another group of three plants of each variety was grown under 3000 lux illumination for 48h and a temperature of 16°C, 24°C,or 32°C. Leaf samples of each variety from each treatment were collected and immediately frozen in liquid nitrogen and subsequently stored at -80°C until use for RNA extraction and mRNA abundance assessment.

Significant differences were analyzed by one-way ANOVA using SPSS 19.0 (IBM Corp., Armonk, NY, USA). Daylength and temperature of each sampling day were also recorded. Correlation between plant growth rate or mRNA abundance of genes of the two varieties and daylength or temperature was analyzed using double factor correlation analysis and one-sided *t*-test, respectively, both available in SPSS 19.0 (IBM Corp., USA). Correlation between mRNA abundance of genes in the two varieties and daylength or temperature was also analyzed. A linear chart of the obtained data was constructed using GraphPad Prism 5 software (GraphPad Software, Inc., San Diego, CA, USA).

### Screening and bioinformatic analysis of candidate genes

After these sequencing reads were trimmed, paired-end reads were assembled by Trinity [16] as a single dataset (reference transcriptome) that was then annotated using BLASTX. The transcript was deposited in DDBJ/EMBL/GenBank under the accession number GAFF00000000. Differentially expressed genes (DEGs) were identified using Simbiot® platform, and the accuracy of the results was tested[13]. DEGs with absolute foldchange ≥4 and adjusted p-value ≤ 0.05 for DM vs. DS and DS vs. NDS were obtained using R software[17], and a custom script was implemented to identify common DEGs(co-DEGs) and unique DEGs for DM vs. DS and DS vs. NDS. Next, the total DEGs in DM vs. DS and DS vs. NDS were screened for unique genes of DS vs. NDS and unique genes of DM vs. DS, respectively. Some of the unique DEGs were also classified as co-DEGs based on the fold-change value and adjusted p-value. In addition, DEGs associated with drought resistance, insect resistance, and disease resistance, as well as DEGs with the same expression trend in DM vs. DS and DS vs. NDS, were excluded. The co-DEGs, unique DEGs of DM vs. DS, and unique DEGs of DS vs. NDS were confirmed and collectively designated ACDEGs.

ACDEGs were imported into Blast2GO for gene ontology (GO) annotation. The sequences of these genes were submitted to the Kyoto Encyclopedia of Genes and Genomes (KEGG) Automatic Annotation Server (KAAS; http://www.genome.jp/kaas-bin/kaas_main; Version 1.67x) to retrieve KEGG orthology (KO) assignments and KEGG pathways using single-directional best hit assignment method. The function of each ACDEG was determined by submitting the amino acid sequences of the ACDEGs to the UniProt database (http://www.uniprot.org/uniprot/) and consulting the literature. The ACDEGs were classified according to their common functions. Finally, key candidate genes involved in the regulation of alfalfa FD were identified based on foldchange in their expression, p-value, and their function in plant growth and development.

### Assessment of mRNA abundance of key genes and correlation analysis between mRNA abundance of genes and daylength or temperature

The mRNA abundance of 44 candidate genes in Maverick leaves (three samples over a 7-month period, resulting in a total of 21 samples) was detected and analyzed from April to October in 2011. Key genes among the 44 candidate genes were selected based on whether their transcription was regulated by photoperiod or temperature and their role in plant growth and development. The mRNA abundance of six key genes in CUF101 leaves(3 samples per month over a 7-month period, resulting in a total of 21 samples) was detected and analyzed again in 2011. The mRNA levels of these genes in Maverick and CUF101 leaves (3 samples ×7months × 2 varieties = 42 samples) were detected and analyzed again in 2016.

The mRNA abundance of the six key genes in Maverick and CUF101 leaves grown under artificial culture conditions (3 samples × 3 treatments × 2 varieties × 2 factors = 36 samples) was detected and analyzed.

Total RNA in all samples was extracted in strict accordance with the TRIzol method (Invitrogen, Carlsbad, CA, USA). Reverse transcription was performed using a RT kit following the manufacturer’s instructions (Takara Bio, Inc.). RNA content and quality were analyzed in a Nano2000 Ultramicro spectrophotometer (ThermoFisher Scientific, Waltham, MA, USA), and each RNA sample was adjusted to the same concentration.

Quantitative reverse transcription polymerase chain reaction (qRT-PCR) primers for each gene were designed using Primer5.0 software (Premier Biosoft International, Palo Alto, CA, USA) following the established principles of qRT-PCR primer design (S1 Table). mRNA abundance of each gene in all samples was detected using the Roche SYBR Green fluorescent dye method and a Roche Cycler9.0 fluorescent PCR instrument (Roche Diagnostics GmbH, Manheim, Germany). The data were normalized against the expression of the housekeeping gene GAPDH, and the expression was calculated relative to a calibrator samples using the formula 2^−ΔΔCt^; the values were presented as mean±standard deviation[18]. Significant differences were assessed by one-way ANOVA, and the correlation of the mRNA abundance of each gene with daylength or temperature was analyzed using double factor correlation analysis and one-sided *t*-test, respectively, implemented in SPSS 19.0 (IBM Corp., USA). The obtained qRT-PCR data were used to construct a linear chart in GraphPad prism 5 (GraphPad Software, Inc., USA).

## Results

### Analysis of DEGs

The DEGs of DM vs. DS and DS vs. NDS were listed in S1 and S2 Files. A total of 538 genes were identified as DEGs in DM vs. DS, of which 122 were annotated and 416 were not annotated. Further, the expression of 337 DEGs was upregulated and201 DEGs were downregulated in DM vs. DS(S3 File). Similarly, of the 836 DEGs identified in DS vs. NDS,156 were annotated and 680 were not annotated; 545 DEGs were upregulated and 291 DEGs were downregulated (S4 File). 86 genes were co-DEGs of DM vs. DS and DS vs. NDS(S5 File); among these, 78 genes were un-annotated and 8 were annotated (S5 File). After a series of analyses and screening, in total, 489 co-DEGs of DM vs. DS and DS vs. NDS were obtained; of these, 405 genes were un-annotated and 84 genes were annotated. Further, 218genes were unique to DS vs. NDS, with163 genes being un-annotated and 55 genes annotated. Of the354genes unique to DM vs. DS, 290 genes were un-annotated and 64 genes were annotated. Overall, ACDEGs comprised 1,069 genes (S6 File).

### GO enrichment analysis of ACDEGs

**Fig 1:**
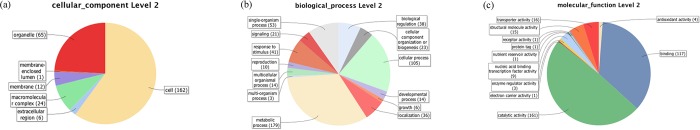
Gene ontology functional classification of key candidate genes. The functional assignments with biological processes, molecular functions, and cellular components are shown based on the number of proteins and the converted corresponding proportion.

The GO enrichment analysis classified most ACDEGs into three significant, broad GO categories: “biological process”, “molecular function” and “cell component”. Within the category “biological process”, the DEGs were assigned to the terms: metabolic process (153 genes), cellular process (113 genes), response to stimulus (37 genes), single-organism process (55 genes), localization (36 genes), cellular component organization or biogenesis (26 genes), signaling (21 genes), developmental process (13 genes), and growth (6 genes). The DEGs assigned to “molecular function” category were enriched in cell (84 genes), organelle (65 genes), macromolecular complex (26 genes), membrane (8 genes), and extracellular region (6 genes). Finally, in the “cell component,” the genes were functionally assigned to catalytic activity (139 genes), binding (95 genes), transporter activity (15 genes), molecular structure (15 genes), and nucleic acid binding transcription factor activity (9 genes) (Fig 1).

### Kyoto Encyclopedia of Genes and Genomes (KEGG) enrichment analysis of ACDEGs

ACDEGs were significantly enriched in 33 pathways (p ≤0.05) according to the KEGG enrichment analysis. Ribosome pathway and metabolic pathways were the two main pathways, and DEGs were significantly enriched in carbohydrate metabolic pathways, such as the starch and sucrose metabolism pathway and the pentose and glucuronate interconversion pathway. The biosynthesis of secondary metabolites, such as cyanoamino acid metabolism, phenylpropanoid biosynthesis, amino sugar and nucleotide sugar metabolism, phenylalanine metabolism, and sesquiterpenoid biosynthesis, was also enriched. Genes involved in protein processing and endoplasmic reticulum pathway coded mainly for heat shock proteins. The RNA transport pathway, ubiquitin-mediated proteolysis pathway, tryptophan metabolism pathway, plant circadian rhythm, and plant hormone signal transduction were also significantly enriched (S7 File).

Based on the results of the KEGG and GO analysis of DEGs and the results of previous studies, 44 key candidate genes may be involved in the regulation of FD (Table 1).

**Table 1:** Key candidate genes involved in regulating alfalfa fall dormancy.

Classification	Number	Tags	Genes	Function [reference]	Expression trend of DM vs. DS	Expression trend of DS vs. NDS
Genes involved in carbohydrate metabolism and transport	1	comp1260_c0	MGDG synthase 3	Synthesis of photosynthetic membranes and chloroplast envelope [19]		Downregulated(-3.53)
	2	comp59094_c0	Beta-D-glucosidase	Catalyzes the hydrolysis of terminal non-reducing residues in beta-D-glucosides with a release of glucose[20]	Downregulated(-2.10)	Upregulated(3.05)
	3	comp25768_c0	UDP-sugar pyrophosphorylase	Catalyzes the conversion of various monosaccharide 1-phosphates to respective UDP-sugars in the salvage pathway[21]	Upregulated(2.14)	Downregulated(-3.19)
	4	comp53413_c0	Ribulose bisphosphate carboxylase/oxygenase (Rubisco) activase	Activates Rubisco	Downregulated(-2.87)	Upregulated(1.01)
Genes regulated by photoperiod and light	5	comp46970_c0	Cryptochrome-2(*CRY-2*)	Inhibits hypocotyl elongation [22] and stem and root growth[23]	Upregulated(3.50)	
	6	comp1262167_c0	Protein FAR-RED IMPAIRED RESPONSE 1(*FAR1*)	Positively regulates chlorophyll biosynthesis via the activation of *HEMB1* gene expression[24]; activates transcription; elongates hypocotyls and reduces expansion of cotyledons[25]		Upregulated(2.23)
	7	comp38709_c0	GATA transcription factor 12(*GATA12*)	Activates transcription, regulates some light-responsive genes and circadian rhythm[26–28]	Upregulated(2.40)	Downregulated(-2.41)
	8	comp12163_c0	Serine protease *SPPA*, chloroplastic	the light-dependent degradation of antenna and photosystem II in chloroplasts[29, 30];	Downregulated(-2.22)	Upregulated(2.23)
	9	comp46405_c0	Protein HEADING DATE 3A	Regulates dormancy [31]	Downregulated(-1.72)	Upregulated(4.38)
	10	comp56985_c0	Granule-bound starch synthase 1(*GBSS1*)	Regulated by photoperiod, may be accompanied by abolition of expression of starch synthesis genes [32]	Downregulated(-2.69)	Upregulated(5.74)
Phytohormone	11	comp36708_c0	Probable indole-3-pyruvate monooxygenase *YUCCA3*	Participates in indoleacetic acid (IAA) synthesis[33]	Upregulated(3.40)	Downregulated(-1.35)
	12	comp343499_c0	Methylesterase17	Hydrolyzes conjugates of IAA	Downregulated(-1.72)	Upregulated(3.38)
	13	comp522282_c0	IAA-amino acid hydrolase ILR1-like 1	Hydrolyzes conjugates of IAA[34]		Upregulated(2.36)
	14	comp15714_c0	Auxin response factor 6(*ARF6*)	Activates transcription, participates in transcriptional regulation of a variety of biological processes related to growth and development[35, 36]		Downregulated(-2.74)
	15	comp57456_c0	ABA receptor *PYL8*	Positively regulates abscisic acid (ABA) signaling pathway[37–40]	Upregulated(2.66)	
	16	comp61323_c0	Protein early responsive to dehydration 15	Negatively regulates ABA responses and mediates stress-related ABA signaling[41, 42]	Upregulated(2.05)	
Transportation	17	comp46282_c0	ATP-binding cassette (ABC) transporter C family member 7	Involved in the response to biotic stress		Upregulated(2.36)
	18	comp10285_c0	ABC transporter B family member 11	Essential regulator of plant growth	Upregulated(2.55)	
	19	comp70285_c0	Monosaccharide-sensing protein 3	Involved in direct growth and differentiation or flexible response to changing environmental conditions (UniProt: Q9SD00)	Upregulated(2.13)	
	20	comp40098_c0	Probable ion channel SYM8	A calcium channel[43]	Upregulated(3.15)	
	21	comp343967_c0	Putative phospholipid-transporting ATPase 7	Transports phospholipids (UniProt: Q9LVK9)	Upregulated(3.15)	Downregulated(-3.32)
	22	comp928260_c0	Ras-related protein *RABH1d*	Involved in protein transport(UniProt: Q9SID8)	Downregulated(-0.83)	Upregulated(2.23)
	23	comp1120261_c0	Oligopeptide transporter 6	Translocates tetra- and pentapeptides[44, 45]	Upregulated(2.40)	Downregulated(-2.41)
Cell cycle, division, and growth	24	comp33056_c0	Cyclin-D5-2	Mediates the linking of extracellular and developmental signals to the cell cycle[46]		Downregulated(-3.60)
	25	comp67791_c1	Anaphase-promoting complex (APC)subunit 1	Marks target cell cycle proteins for degradation by 26s proteasome; involved in eukaryotic cell reproduction[47–49]	Downregulated(-1.27)	Upregulated(5.11)
	26	comp686013_c0	Protein S-acyltransferase 24 (*PAT24*)	Involved in cell growth regulation [50]; affects root hair formation and growth [51, 52]	Upregulated(2.20)	
	27	comp32326_c0	Monocopper oxidase-like protein *SKU5*	Possibly involved in directional growth processes and cell wall expansion[53]	Upregulated(2.20)	
	28	comp672512_c0	Prohibitin-3, mitochondrial	Loss of function of the homologs AtPHB3 causes mitochondrial welling, decreases meristematic cell production, increases cell division time, and reduces cell expansion rates, leading to severe growth retardation[54]	Downregulated(-0.83)	Upregulated(2.36)
	29	comp887354_c0	Kinesin-4	Involved in cell cycle[55] and cell division[56–61].	Downregulated(-2.92)	Upregulated(2.23)
	30	comp37666_c0	Structural maintenance of chromosomes protein 3(*SMC3*)	Essential protein for plant viability, required for cell division during embryogenesis, increased expression accelerates cell cycle [62, 63]		Upregulated(2.23)
Genes regulating transcription	31	comp424089_c0	Probable histone H2A.2	Plays a central role in transcription regulation, DNA repair, DNA replication, and chromosomal stability. Involved in gene regulation[64]		Upregulated(2.36)
	32	comp57595_c0	Histone H3.3		Upregulated(6.23)	Downregulated(-4.08)
	33	comp391402_c0	MADS-box transcription factor PHERES 1	Regulates dormancy[31, 65]	Downregulated(-3.20)	Upregulated(3.12)
	34	comp50413_c1	Squamosa promoter-binding protein 1(*SBP1*)	Activates transcription, and involved in leaf development, vegetative phase change, etc. [66, 67]	Upregulated(3.97)	
Ubiquitination	35	comp53272_c0	Probable mediator of RNA polymerase II transcription subunit 37c	Coactivator regulates transcription of nearly all RNA polymerase II-dependent genes [UniProt: Q9LHA8—MD37C_ARATH]		Downregulated(-6.76)
	36	comp538610_c0	BTB/POZ domain-containing protein At1g67900	Mediates transcriptional repression [UniProt: Q9C9V6—Y1790_ARATH]	Upregulated(2.40)	
	37	comp933338_c0	BTB/POZ domain-containing protein At3g19850	Mediates transcriptional repression [UniProt: Q9LT24—Y3985_ARATH]	Upregulated(2.40)	
	38	comp29926_c0	F-box only protein 6	Substrate-recognition component of some SCF-type E3ubiquitin-ligase complexes, participates in regulation of auxin-mediated signaling pathway, leaf vascular tissue pattern formation, and simple leaf morphogenesis[68, 69]		Upregulated(5.50)
	39	comp655910_c0	UBX domain-containing protein 7	Acts in many cellular events such as ubiquitin-dependent degradation and membrane fusion[70]	Upregulated(2.87)	Downregulated(-3.00)
	40	comp909276_c0	Putative E3 ubiquitin-protein ligase *LIN*	Catalyzes polyubiquitination with ubiquitin-conjugating enzyme E2 UBC8 in vitro, involved in plant C/N response and early steps of the plant defense signaling pathway[71]	Upregulated(2.40)	
	41	comp395328_c0	E3 ubiquitin-protein ligase *UPL6*	Mediates ubiquitination and subsequent proteasomal degradation of target proteins [UniProt:Q8RWB8—UPL6_ARATH]	Downregulated(-2.96)	Upregulated(1.68)
Receptor kinases	42	comp70176_c0	Wall-associated receptor kinase(*WAK*)5; Wall-associated receptor kinase-like (*WAKL*)2	Involved in cell expansion, elongation[72, 73]	Upregulated(2.36)	
	43	comp41596_c0	*WAK3*			Downregulated(-3.00)
	44	comp403595_c0	Serine/threonine-protein kinase *Nek1*	Involved in sensing and repair of DNA strand breaks at the G1-S and G2-M transitions[74]	Upregulated(2.40)	Downregulated(-2.41)

DM and DS: Leaf samples of the fall-dormant alfalfa variety collected in May and September, respectively; NDS: leaf samples of the non-dormant alfalfa variety collected in September.

### Daylength, temperature, leaf area, and plant height of the Maverick variety from April to October

**Fig 2:**
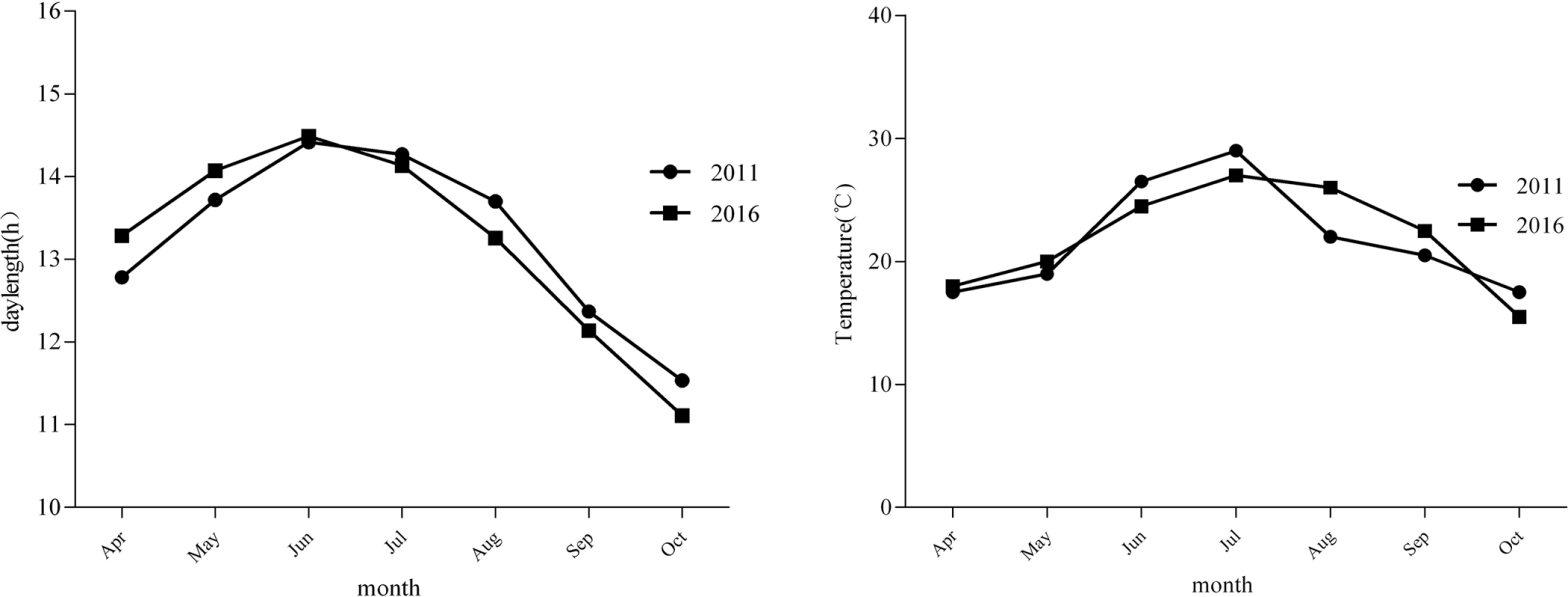
Daylength and temperature from April to October.

Daylength and temperature first increased and then decreased from April to October: the longest day was in June, and the highest temperature was measured in July (Fig 2).

**Fig 3:**
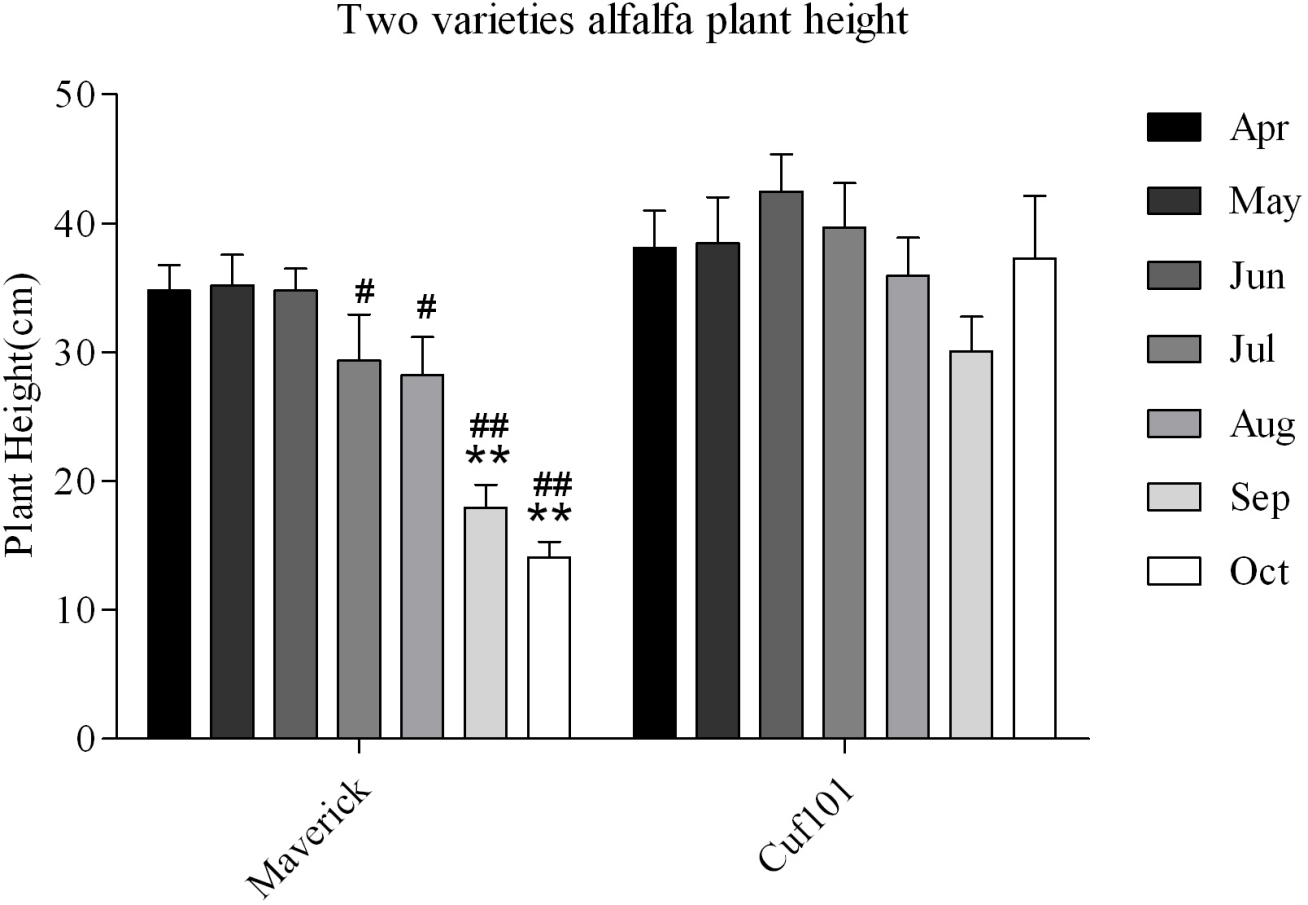
Plant height of Maverick and CUF101varieties from April to October. The difference in plant height in Maverick is marked with *. The difference in plant height between Maverick and CUF101 is marked with #. (*, #, p < 0.05; **, ## p< 0.01).

**Fig 4:**
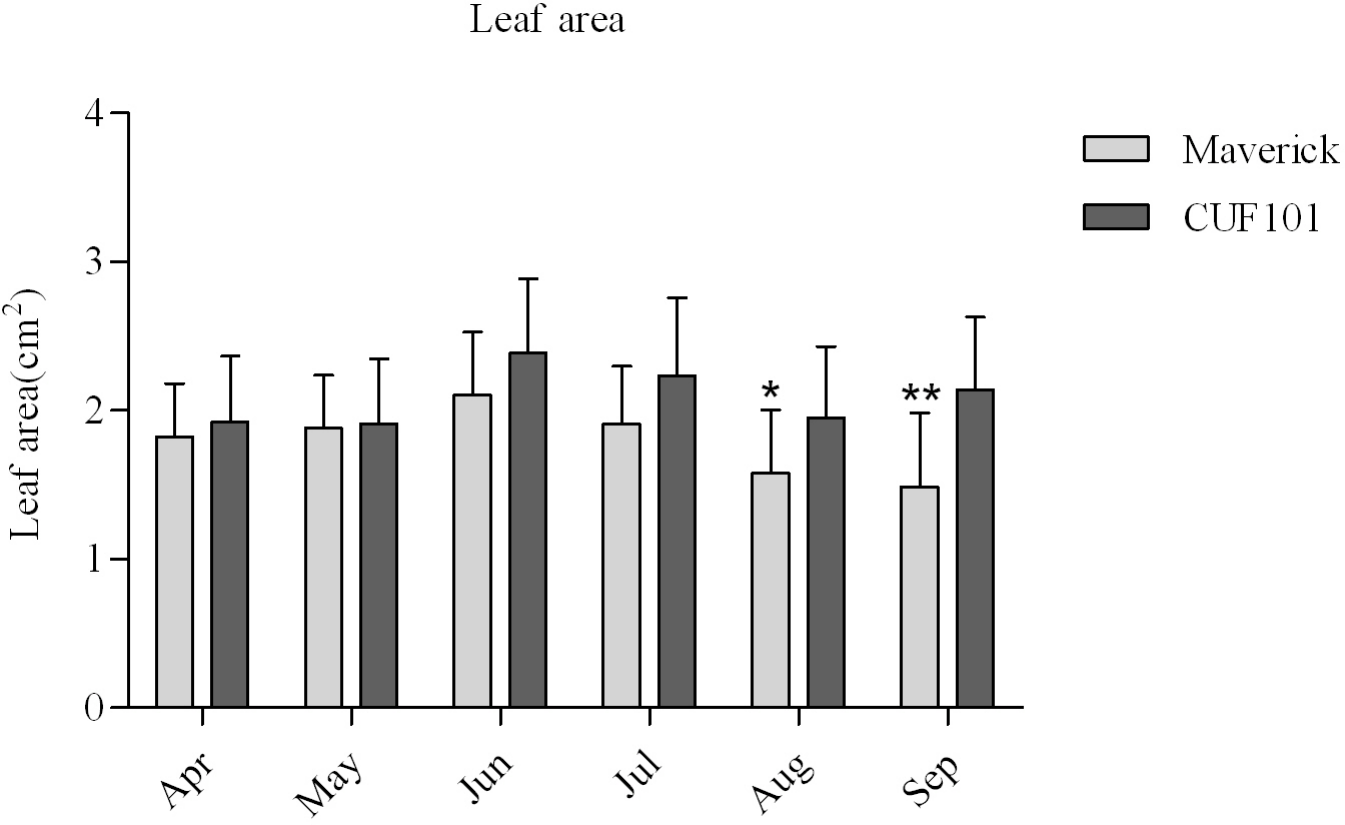
Leaf area of Maverick and CUF101 varieties from April to October. * and **indicate significant difference at p < 0.05 and p < 0.01, respectively.

The plant height of Maverick did not differ significantly between April and August, but it decreased significantly in September and October as compared with that from April to August. The plant height of CUF101 did not differ significantly between July and October, and it was significantly higher than that of Maverick in the same period (Fig 3). The leaf area of Maverick was smaller than that of CUF101 from April to September, and the difference in leaf area between the two varieties reached its maximum in September. There was no significant difference in the leaf area of CUF101 from June to September, whereas the leaf area of Maverick decreased from June to September. In addition, the leaf area of Maverick in August and September was significantly smaller than that in June and July (Fig 4).

### mRNA abundance of four ACDEGs from April to October in the two alfalfa varieties

**Fig 5:**
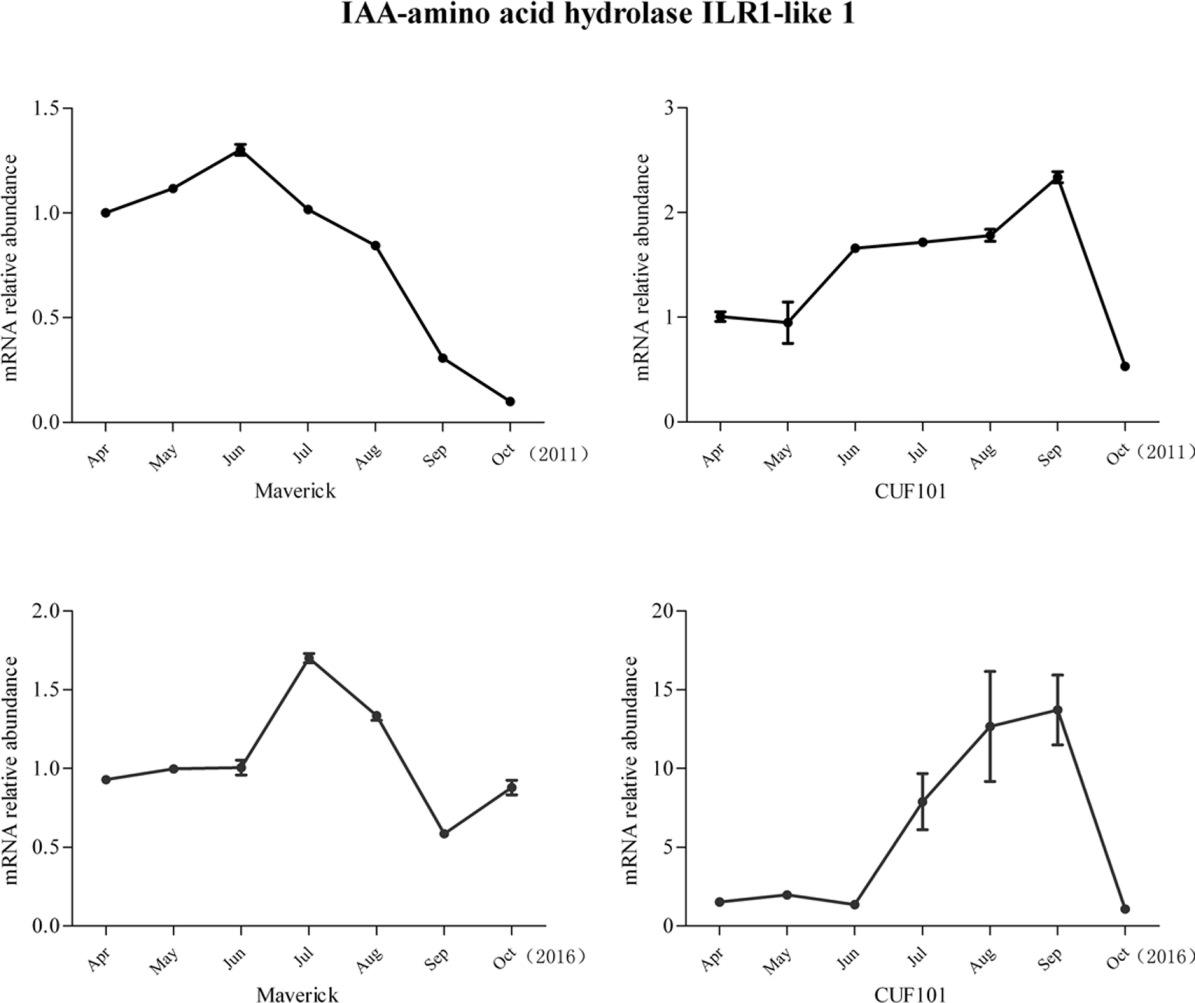
mRNA abundance of indoleacetic acid (IAA)-amino acid hydrolase ILR1-like 1 in Maverick and CUF101 varieties from April to October in 2011 and 2016. * and ** indicate significant difference between August-September and June-July at p<0.05 and p<0.01, respectively.

**Fig 6:**
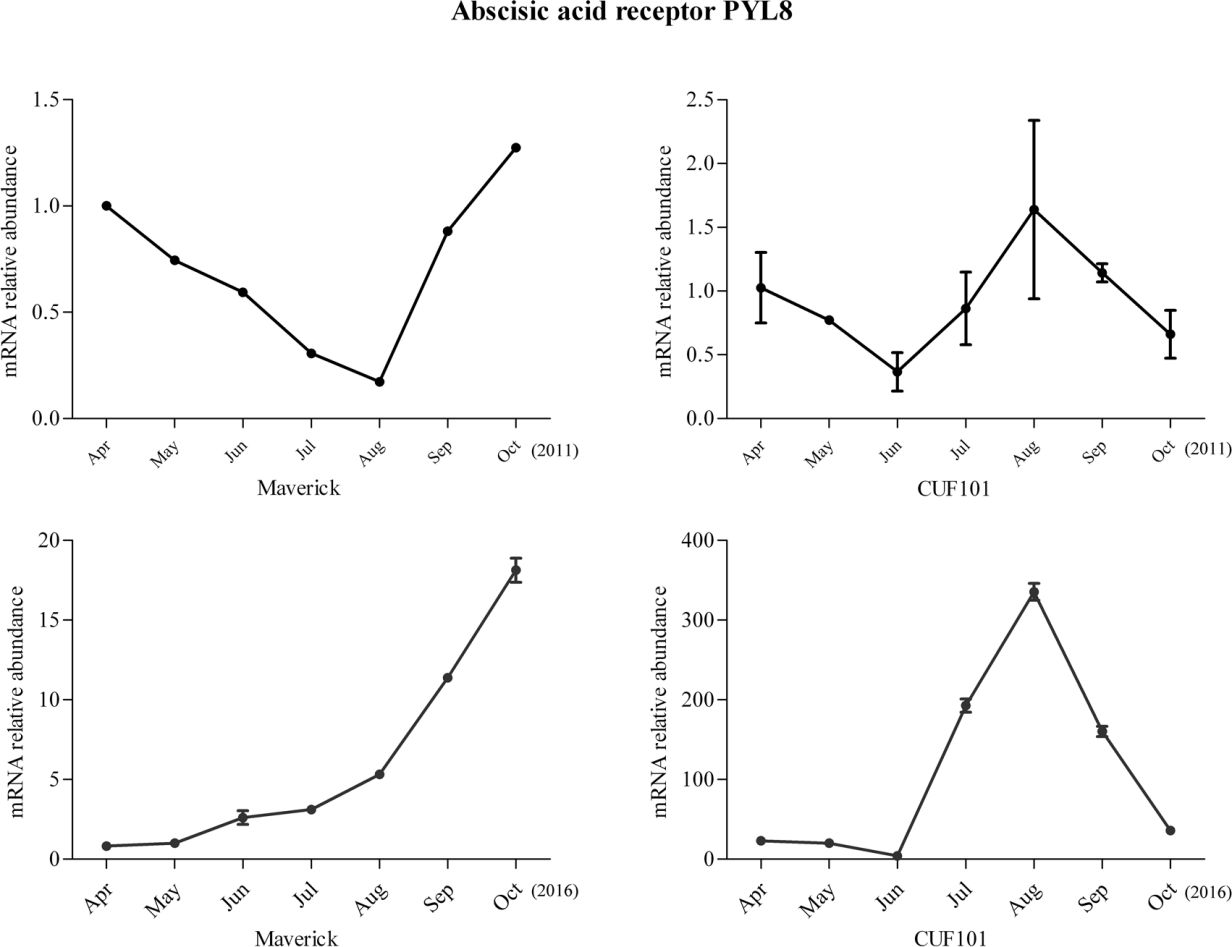
mRNA abundance of the abscisic acid receptor PYL8 in Maverick and CUF101 varieties from April to October in 2011 and 2016. ** indicates significant difference between September-October and August at p<0.01.

**Fig 7:**
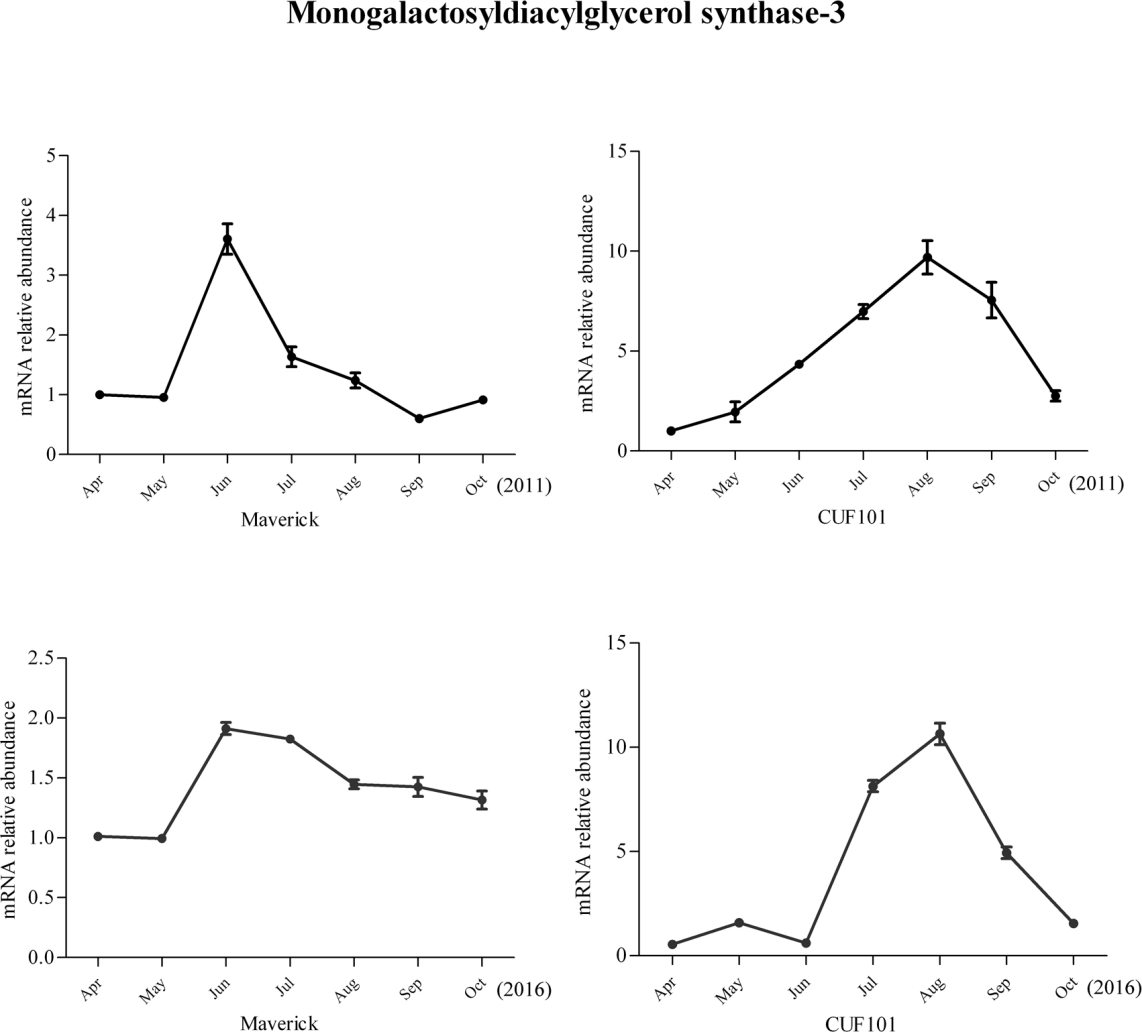
mRNA abundance of monogalactosyldiacylglycerol synthase-3 in Maverick and CUF101 varieties from April to October in 2011 and 2016. * and ** indicate significant difference between July, August, September, and June at p<0.05 and p<0.01, respectively.

**Fig 8:**
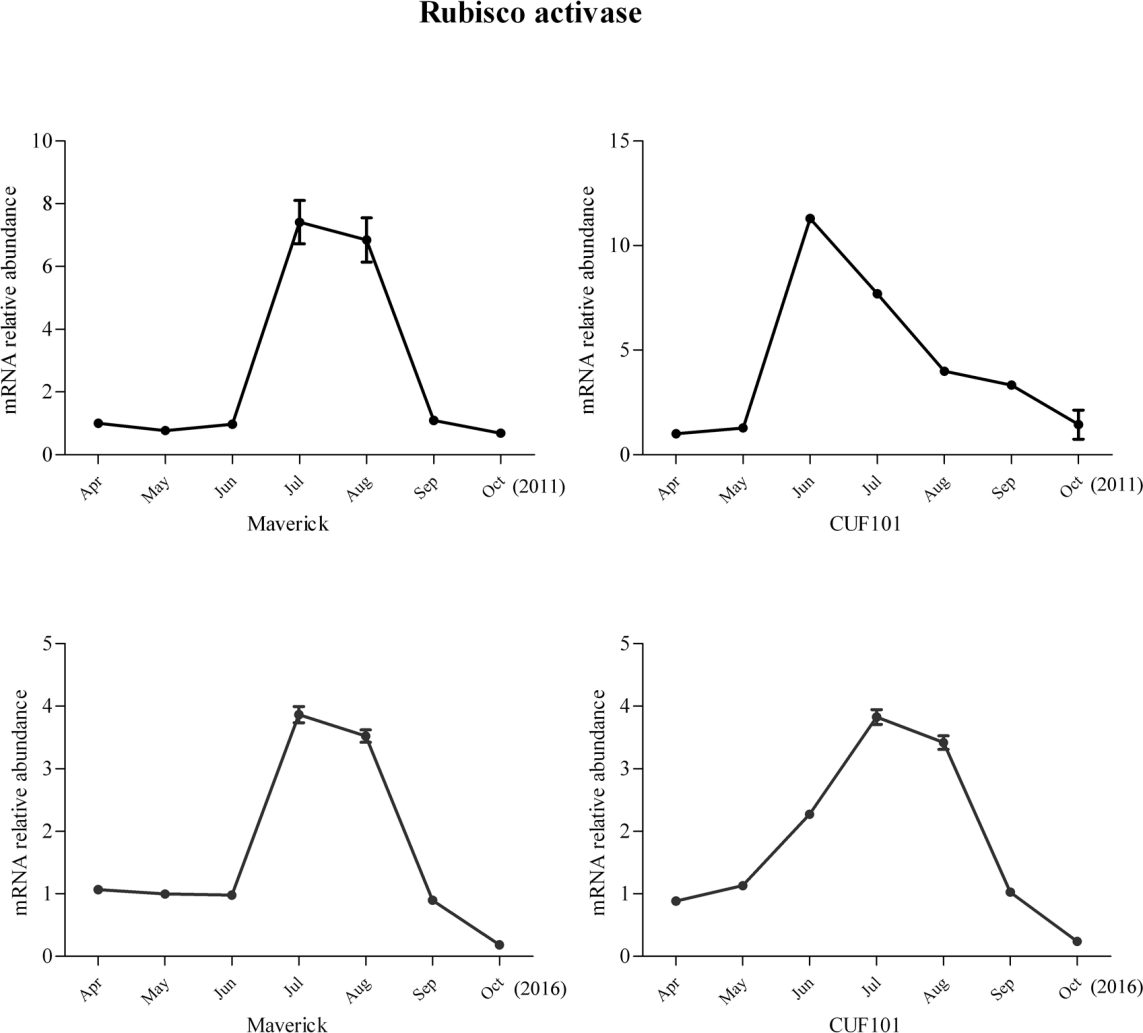
mRNA abundance of Rubisco activase in Maverick and CUF101varieties from April to October in 2011 and 2016. ** indicates significant difference between September, October, July, and August at p<0.01.

Among the 44 DEGs, mRNA abundance of six DEGs was significantly correlated with daylength and temperature in 2011. However, the comparison of the data between 2011 and 2016 revealed that the change in mRNA abundance of one gene in both varieties differed between the two sampling years and the change in mRNA levels of another gene followed the same trend in both varieties and thus was not significantly different between the two varieties. Therefore, these two genes were excluded from further analysis. The mRNA profiles of IAA-amino acid hydrolase ILR1-like 1(ILR-like 1), ABA receptor PYL8(PYL8), monogalactosyldiacylglycerol (MGDG) synthase-3(MGDGS-3), and Ribulose bisphosphate carboxylase/oxygenase (Rubisco) activase are discussed herein. The mRNA profiles of the four genes exhibited the same regular, specific trends from April to October in 2011 and 2016, with significant differences detected between Maverick and CUF101 (Figs 5–8). The mRNA data of other genes are not shown.

The change in mRNA abundance of ILR1-like 1 and the PYL8 followed the opposite trends in the autumn. Thus, the mRNA abundance of ILR1-like 1 decreased in Maverick but increased in CUF101 from June to September, whereas the mRNA abundance of the PYL8 increased in Maverick but decreased in CUF101 in the same period. In Maverick, the mRNA abundance of ILR1-like 1 in August to October was significantly lower than that in June and July. In CUF101, the abundance of the same mRNA was significantly higher in July to September than it was in June. The mRNA abundance of the PYL8 was significantly higher in September and October in Maverick but significantly lower in CUF101 as compared to those in August(Figs 5 and 6).

The mRNA abundance of MGDGS-3 in the two varieties first increased and then decreased starting from June (in Maverick) and August (in CUF101). In Maverick, the mRNA abundance of MGDGS-3 was significantly lower from July to October than it was in June, but in CUF101, it was significantly higher from July to September compared with that in June. The mRNA abundance of MGDGS-3 from July to September was significantly lower in Maverick compared to that in CUF101 (Fig 7). Similarly, the abundance of the Rubisco activase mRNA increased initially in both varieties, which was followed by a decrease from July onwards. In both alfalfa varieties, its mRNA abundance in September and October was significantly lower than that in July and August(Fig 8).

### mRNA profiles of four DEGs in leaves of the two alfalfa varieties under artificial growth conditions

**Fig 9:**
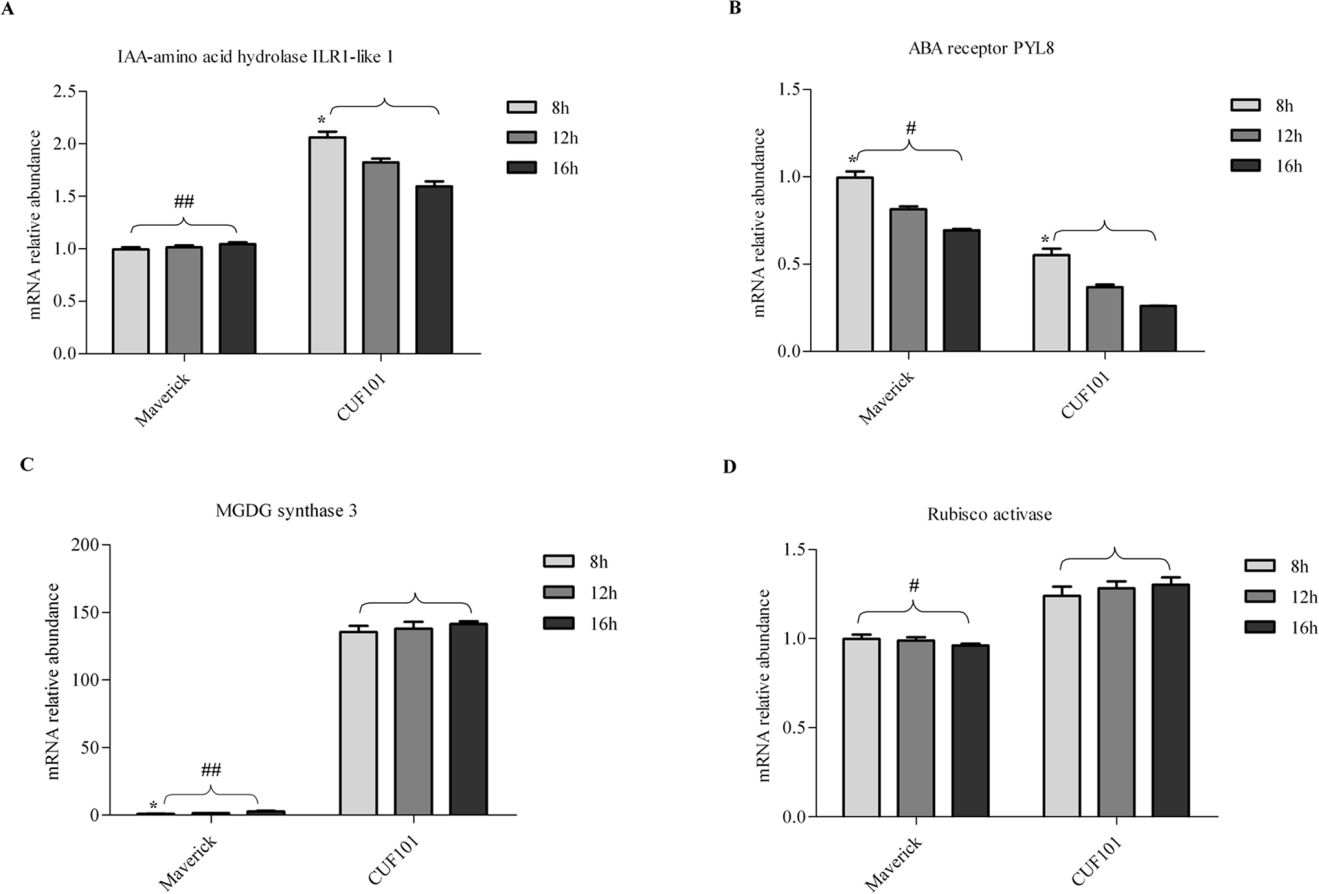
mRNA abundance of four differentially expressed genes (DEGs) in Maverick and CUF101 varieties grown under 8h to 16h daylength and artificial growth conditions. Significant difference in the mRNA content of DEGs at 8h,12h, and 16h of illumination in each variety is marked with *. Significant difference in mRNA content between the two varieties is indicated with # (*, #, p<0.05; **, ##,p<0.01). Error bars indicate standard deviation (SD).

The change in mRNA abundance of ILR1-like 1 in Maverick followed the opposite trend to that in CUF101 with increasing illumination from 8h to 16h. Thus, its mRNA abundance in Maverick gradually increased with increasing illumination from 8h to 16h and was significantly less than that in CUF101 (Fig 9A).

The mRNA abundance of the PYL8 in the two varieties gradually decreased with increasing illumination from 8h to 16h and was significantly greater than that in CUF101 (Fig 9B).

As the illumination increased from 8 h to 16 h, the mRNA abundance of MGDGS-3 increased and that of the rubisco activase mRNA was not significantly altered. The levels of both genes of mRNA in Maverick were significantly lower than those in CUF101 (Fig 9C and 9D).

**Fig 10:**
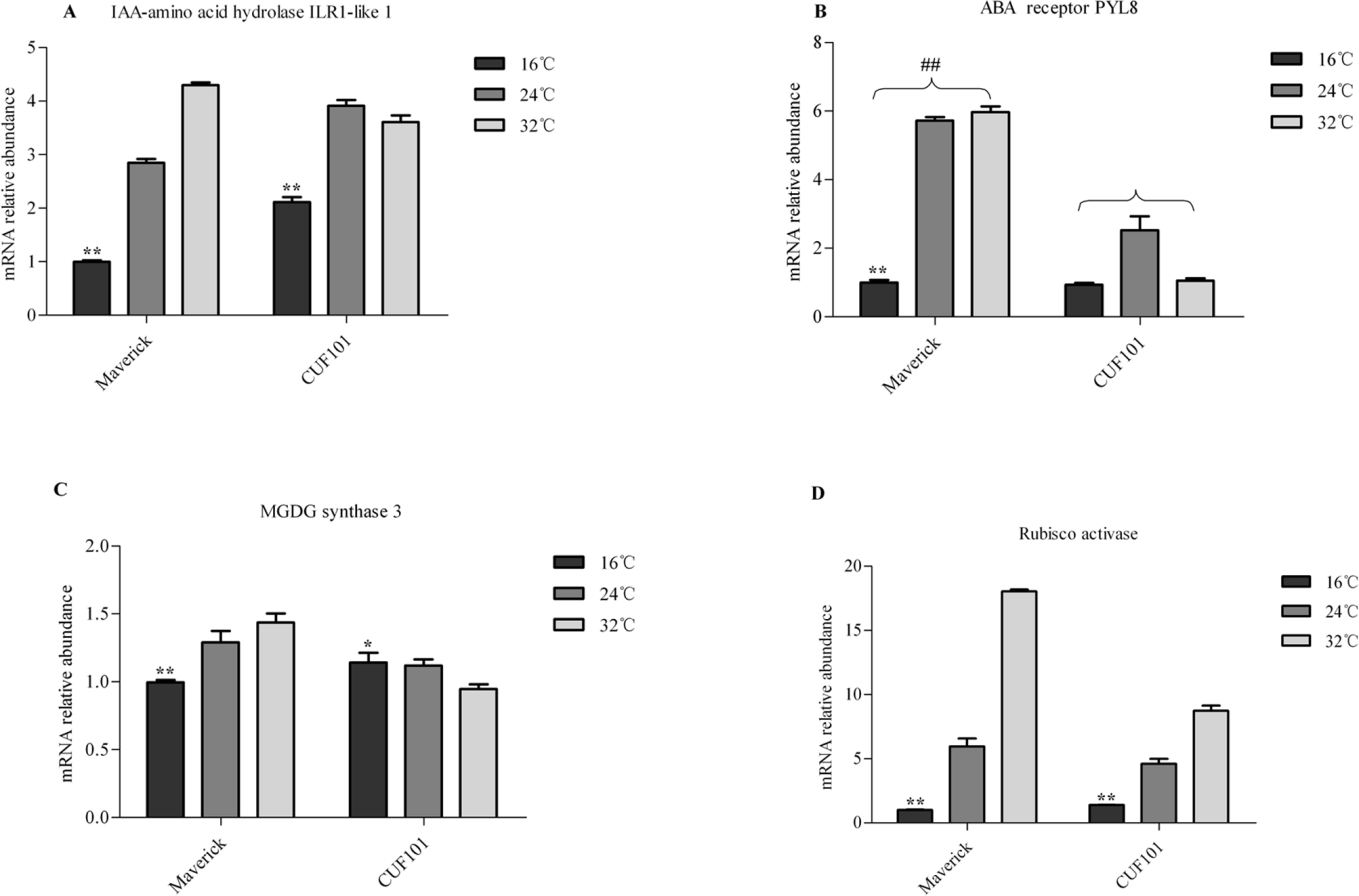
mRNA abundance of four differentially expressed genes (DEGs) in Maverick and CUF101 varieties grown under artificial growth conditions at different temperatures (from 32°C to 16°C). Significant difference in mRNA content of DEGs between 16°C, 24°C and 32°C for each variety is marked with *. Significant difference in mRNA content between the two varieties is marked with # (*, #, p<0.05; **, ##, p<0.01). Error bars indicate standard deviation (SD).

The changes in mRNA abundance of the ILR1-like 1 and the PYL8 in the same variety followed the same trend as temperature increased from 16°C to 32°C.Thus, their mRNA abundance gradually increased from 16°C to 32°C in Maverick, whereas in CUF101, the abundance of the two mRNAs at 24°C was greater than that at 16°C and 32°C (Fig 10A and 10B). Similarly, the abundance of the Rubisco activase mRNA in the two varieties gradually increased from 16°C to 32°C, but it reached higher levels in Maverick than in CUF101 (Fig 10C). In contrast, the levels of the MGDGS-3 mRNA gradually increased in Maverick but decreased in CUF101 with increasing temperature from 16°C to 32°C (Fig 10D).

### Correlation analysis of plant growth rate, mRNA abundance of genes, and daylength or temperature under natural conditions

The growth rate of Maverick was significantly correlated with daylength, but not with temperature, whereas the growth rate of CUF101 was not significantly correlated with either daylength or temperature (Table 2).

**Table 2:** Correlation of the growth rate with daylength and temperature in Maverick and CUF101 varieties.

	Maverick growth rate		CUF101 growth rate	
	Pearson correlation	Significance	Pearson correlation	Significance
Daylength	0.858**	0.007	0.593	0.080
Temperature	0.107	0.410	0.445	0.158

**represent significant correlation at 0.01 level.

The mRNA abundance of ILR1-like 1 in Maverick was significantly positively correlated with daylength and moderately positively correlated with temperature in both varieties in the two experimental years. In contrast, the abundance of the ILR1-like 1 mRNA in CUF101 had no significant correlation with daylength and temperature (Tables 3 and 4).

**Table 3:** Correlation between mRNA abundance in the leaves of Maverick and CUF101 varieties and daylength in 2011 and 2016.

	Daylength							
	Maverick				CUF101			
	2011		2012		2011		2012	
	Pearson correlation	Significance	Pearson correlation	Significance	Pearson correlation	Significance	Pearson correlation	Significance
IAA-amino acid hydrolase ILR1-like 1	0.975**	0.000	0.700*	0.040	0.232	0.308	-0.137	0.385
Abscisic acid receptor PYL8	-0.664	0.052	-0.92**	0.001	-0.184	0.346	-0.035	0.47
MGDG synthase 3	0.610	0.073	0.318	0.244	0.012	0.49	0.054	0.454
Rubisco activase	0.294	0.261	0.438	0.163	0.579	0.086	0.604	0.075

IAA: indoleacetic acid; MGDG: monogalactosyldiacylglycerol.

*and **represent significant correlation at 0.05 and 0.01 level, respectively. IAA: indoleacetic acid; MGDG: monogalactosyldiacylglycerol.

**Table 4:** Correlation between mRNA abundance in the leaves of Maverick and CUF101 varieties and temperature in 2011 and 2016.

	Temperature							
	Maverick				CUF101			
	2011		2012		2011		2012	
	Pearson correlation	Significance	Pearson correlation	Significance	Pearson correlation	Significance	Pearson correlation	Significance
IAA-amino acid hydrolase ILR1-like 1	0.491	0.131	0.627	0.066	0.545	0.103	0.599	0.078
Abscisic acid receptor PYL8	-0.726*	0.032	-0.430	0.167	0.192	0.34	0.654	0.055
MGDG synthase 3	0.665	0.051	0.713*	0.036	0.506	0.123	0.714*	0.035
Rubisco activase	0.612	0.072	0.807*	0.014	0.899**	0.003	0.926**	0.0015

IAA: indoleacetic acid; MGDG: monogalactosyldiacylglycerol.

* and ** represent significant correlation at 0.05 and 0.01 level, respectively.IAA: indoleacetic acid; MGDG: monogalactosyldiacylglycerol.

The mRNA abundance of the PYL8 was significantly negatively correlated with daylength and moderately negatively correlated with temperature in Maverick, but had no significant correlation with the two parameters in CUF101 (Tables 3 and 4).

In both varieties, the abundance of the MGDGS-3 mRNA was significantly positively correlated with temperature, and that of the Rubisco activase was moderately positively correlated with temperature; neither of the two was correlated with daylength (Tables 3 and 4).

### Correlation analysis of mRNA abundance of genes and daylength or temperature under artificial growth conditions

The correlation of the mRNA abundance of ILR1-like 1 and that of Rubisco activase was in the two varieties with illumination time was opposite. The mRNA abundance of ILR1-like 1 and Rubisco activase in Maverick was significantly positively and negatively correlated with illumination time, respectively. Correlation of the mRNA abundance of PYL8 and MGDGS-3 in Maverick and CUF101 with illumination time was same. The mRNA abundance of PYL8 was significantly negatively correlated with illumination time. The mRNA abundance of MGDGS-3 was significantly positively correlated with illumination time. (Table 5).

**Table 5:** Correlation between mRNA abundance in the leaves of Maverick and CUF101 varieties and illumination time under artificial growth conditions.

	illumination time			
	Maverick		CUF101	
	Pearson correlation	Significance	Pearson correlation	Significance
IAA-amino acid hydrolase ILR1-like 1	0.812**	0.008	-0.984**	0
Abscisic acid receptor PYL8	-0.987**	0	-0.981**	0
MGDG synthase 3	0.767*	0.016	0.666*	0.05
Rubisco activase	-0.756*	0.019	0.617	0.077

* and ** represent significant correlation at 0.05 and 0.01 level, respectively.

IAA: indoleacetic acid; MGDG: monogalactosyldiacylglycerol.

Correlation of the mRNA abundance of MGDGS-3 in Maverick and CUF101 with temperature was opposite, the mRNA abundance of MGDGS-3in Maverick was significantly positively correlated with temperature. Correlation of the mRNA abundance of other three genes in Maverick and CUF101 with temperature was same, their mRNA abundance was significantly positively correlated with temperature (Table 6).

**Table 6:** Correlation between mRNA abundance in the leaves of Maverick and CUF101 varieties and temperature under artificial growth conditions.

	Temperature			
	Maverick		CUF101	
	Pearson correlation	Significance	Pearson correlation	Significance
IAA-amino acid hydrolase ILR1-like 1	0.997**	0	0.771*	0.015
Abscisic acid receptor PYL8	0.886**	0.001	0.062	0.874
MGDG synthase 3	0.947**	0	-0.842**	0.004
Rubisco activase	0.971**	0	0.996**	0

* and ** represent significant correlation at 0.05 and 0.01 level, respectively.

IAA: indoleacetic acid; MGDG: monogalactosyldiacylglycerol.

Thus, in Maverick, the correlation of the mRNA abundance of the PYL8 and Rubisco activase with illumination time and temperature followed the opposite trends, whereas that of the mRNA abundance of the ILR1-like 1 and MGDGS-3with illumination time and temperature was the same (Tables 5 and 6). In CUF101, the correlation of the mRNA abundance of MGDGS-3 with illumination time and temperature was the opposite, but that of the ILR1-like 1,PYL8, and Rubisco activase was the same (Tables 5 and 6).

## Discussion

At the whole-plant level, communication between various organs in a plant is involved in the coordination of growth processes at different organizational levels. Thus, growth of individual organs is regulated by long-distance communication from other organs[75]. FD is the overall growth performance of the whole plant during special phases and environment conditions. Leaf plays a key role in the growth of the whole plant through photosynthesis, respiration, transpiration, and other basic functions involved in plant growth, and changes in those functions affect the growth of the whole alfalfa plant. Thus, the leaf plays an important role in alfalfa FD. The comparison of the leaf area in different months between fall-dormant and non-dormant alfalfa indicated that this parameter may be used as an important index of differences between various alfalfa varieties in terms of FD.

In the present study, beside a few annotated DEGs similar to dormancy-regulation genes, the GO and KEGG analysis revealed that ACDEGs play roles in basic biological processes and pathways involved in the regulation of plant growth and development, such as in the response to macro environmental factors (e.g., light, photoperiod, temperature), the photosynthesis and respiration of leaves, and the regulation of phytohormones. Therefore, these genes are expected to be involved in the regulation of FD.

Temperature and especially photoperiod are important environmental factors that regulate FD[6]. In the present study, the growth of Maverick was induced by daylength, whereas FD of Maverick was accompanied by the shortening of daylength. These findings confirm that photoperiod is a key environmental factor regulating FD in alfalfa and thus corroborate previous study[6]. Under artificial growth conditions, the expressions of the ILR1-like 1 and Rubisco activase in Maverick and CUF101 were oppositely affected by illumination, and expression of MGDGS-3 in Maverick and CUF101 was oppositely affected by temperature. Comparison of the correlations between mRNA abundance of the four genes and the two environmental factors (daylength and temperature) under natural and artificial growth conditions showed that the expression of ILR1-like 1 is upregulated by daylength and temperature, whereas in CUF101, it is mainly affected by temperature. The expression of the PYL8 in Maverick is affected by daylength, and that of MGDGS-3 is affected by daylength and temperature in Maverick or by daylength in CUF101. Temperature was the main factor affecting the levels of Rubisco activase in the two cultivars. Therefore, these results suggest that the responses of Maverick and CUF101 to changes of daylength and temperature are different.

Leaf is an important organ involved in phytohormone synthesis. Phytohormones synchronize developmental processes by adjusting plant growth in response to intrinsic and environmental cues [76, 77]. Thus, IAA and ABA play key roles in plant growth and development[78], indole-3-pyruvate monooxygenase YUCCA3, methylesterase 17, and ILR1-like 1 in ACDEGs participate in IAA synthesis and activation[33–36], and PYL8 and early responsive to dehydration 15 in ACDEGs are associated with the ABA signaling pathway[42, 79]. However, the present findings suggest that downregulation of ILR1-like 1 transcription and upregulation of PYL8 transcription were most likely the only factors involved in alfalfa FD in response to shortened photoperiod and a drop in temperature.

Auxins, such as IAA, possess various functions including induction of cell) elongation and cell division, which are important for plant growth and development[80]. ILR1-like 1 releases IAA by hydrolyzing specific amino acid conjugates of the plant growth regulator IAA [34]. The GO analysis showed that ILR1-like 1 was involved in metabolic processes resulting in cell growth(GO:0008152). Decreased expression of ILR1-like 1 in Maverick observed after reduction in daylength and drop in temperature as the summer transitioned into autumn indicates that its expression was regulated by daylength and temperature. However, in CUF101, the expression of ILR1-like 1 increased with shorter daylength in the autumn. Our previous study showed that, compared with semi- and non-dormant alfalfa, the decrease in IAA content in fall-dormant alfalfa was greater and more rapid in response to daylength shortening under artificial growth conditions[14]. In addition, plant height decreased in the fall-dormant alfalfa but remained constant in non-fall-dormant alfalfa from summer to autumn. Therefore, it is speculated that the reduction of ILR1-like 1 participates in alfalfa FD; specifically, its decrease leads to an increase in amino acid conjugates of IAA and a reduction of IAA levels with daylength shortening. Biological activity of an IAA conjugate is opposite from the function of the IAA itself[81]. A previous study showed that exogenous IAA-Ala treatment of tomato inhibits IAA-induced shoot growth and root initiation[82], which explains the observed increase in amino acid conjugates of IAA during IAA-inhibited plant growth.

ABA-mediated signaling plays a critical role in the responses of plants to environmental stresses[83]. In the present study, PYL8 was involved in plant hormone signal transduction (KO 04075). The expression of the PYL8 in Maverick was significantly negatively regulated by daylength and it increased with shortening of daylength. Furthermore, our previous study showed that, compared with semi- and non-dormant alfalfa, ABA content in fall-dormant alfalfa increased more rapidly and by a greater amount in response to daylength shortening under artificial growth conditions, reaching significantly higher levels compared to those in non-dormant alfalfa when the daylength was 13h or less[14]. In addition, plant height of the fall-dormant alfalfa decreased from summer to autumn, whereas that of the non-dormant alfalfa was not reduced. Previous studies demonstrated that ABA is associated with bud dormancy[84], inhibition of seed germination, and prevention of loss of seed dormancy[85, 86]. The PYL8, which is required for ABA-mediated responses (such as stomatal closure and germination inhibition), inhibits the activity of group-A protein phosphatases type 2C when activated by ABA, thus positively regulating the ABA signaling pathway[37–40]. Therefore, it is speculated that increased levels of the PYL8 are involved in alfalfa FD by enhancing the ABA signaling pathway.

Leaf photosynthesis plays a key role in plant growth. MGDG is the most abundant integral lipid in the thylakoid membrane and the photosystem II (PSII) complex[87, 88], which maintains both the linear electron transport process and the photostability of the PSII apparatus[89]. MGDGS-3was found to be involved in the glycolipid biosynthetic process(GO:0009247). The final step of the MGDG biosynthesis is catalyzed by the MGDG synthase[90, 91]. The mRNA abundance of MGDGS-3 in Maverick decreased with shorter illumination time and temperature drop, reaching levels that were significantly lower than those in CUF101 in the autumn; the plant height of Maverick was significantly lower than that of CUF101.Similarly, MGDG-deficient transgenic tobacco plant M18 exhibits retarded growth[89]. Therefore, reduction of MGDGS-3 levels is involved in alfalfa FD. Given that decreased MGDG content in Arabidopsis thaliana and tobacco have been associated with reduction in MGDG synthase levels[89, 92], the same is expected to occur in alfalfa. In addition, reduced MGDG levels reduce thylakoid membrane and the rate of photosynthesis[92, 93]. Therefore, it is suggested that the decrease in MGDG synthase participates in alfalfa FD by reducing leaf photosynthesis in response to temperature drop.

Rubisco(ribulose-1,5-bisphosphate carboxylase/oxygenase) is a key protein in plants. The change in its expression affects the photosynthesis and plant growth by altering the availability of N [94]. Rubisco can be activated by rubisco activase[95]. Previous studies demonstrated that the decrease in the expression of Rubisco activase may lead to the reduction of the photosynthetic rate and plant growth due to reduced activity of Rubisco. In addition, moderately high temperature was found to inhibit Rubisco activase-mediated activation of Rubisco[96]. Therefore, the reduction of Rubisco activase, which trigged by the fall in temperature, significantly reduces the activation of Rubisco or the light-saturated photosynthetic rate[97–99]. Considering that enhanced thermostability of Rubisco activase in *Arabidopsis* has been shown to improve CO_2_ assimilation rates and plant growth under heat stress[100, 101] and as our results showed that the expression of Rubisco activase was positively regulated by temperature, it was expected that the change in the expression of Rubisco activase would affect alfalfa growth. However, the changes in mRNA abundance of rubisco activase followed the same trend in Maverick and CUF101 and its abundance showed no difference between the two varieties, suggesting that rubisco activase is not involved in FD of alfalfa.

## Conclusion

In the present study, 44 important candidate genes likely associated with alfalfa growth and FD were identified. These genes were mainly enriched in the following categories: transduction of light and photoperiod signals and leaf-derived signals (carbohydrates and phytohormones); the process of cell cycle, division, and growth; transcription factors, ubiquitination proteins; receptor kinases; and un-annotated genes. The present work demonstrates that the reduction of ILR1-like 1 and the increase of PYL8 and MGDGS-3 promote alfalfa FD in a response to changes in photoperiod or temperature.

## Supporting information

S1 Table qRT-PCR primers of 37 differentially expressed genes. (DOCX)

S1 File Complete list of differentially expressed genes identified between samples of fall-dormant alfalfa varieties collected in May and September. (CSV)

S2 File Complete list of differentially expressed genes identified between samples of the fall-dormant alfalfa variety and non-fall-dormant alfalfa variety collected in September. (CSV)

S3 File Differentially expressed genes (fold change ≥ 4 and adjusted p-value ≤ 0.05)identified between fall-dormant alfalfa varieties in May and September. (XLS)

S4 File Differentially expressed genes (fold change ≥ 4, adjusted p-value ≤ 0.05) identified between samples of fall-dormant alfalfa variety and non-fall-dormant alfalfa collected in September. (XLS)

S5 File Common differentially expressed genes (fold change ≥ 4, adjusted p-value ≤ 0.05) in samples of fall-dormant alfalfa variety and non-fall-dormant alfalfa collected in September, and differentially expressed genes in samples of fall-dormant alfalfa variety collected in May and September. (XLSX)

S6 File Candidate genes regulating alfalfa fall dormancy (ACDEGs) are identified after discarding the genes for drought resistance, insect resistance, etc. The genes showing the same trends in the expression between fall-dormant alfalfa varieties in May and September were excluded.
(XLSX)

S7 File Pathways of candidate genes regulating alfalfa fall dormancy. (DOCX)

## References

[1] Barnes DK, Smith DM, Stucker RE, Elling LJ. Fall dormancy in alfalfa: A valuable predictive tool [to predict winterhardiness and cultivar adaptation in Minnesota]. Agricultural Reviews & Manuals Arm Nc. 1979.

[2] Larry R. Teuber KLT, Larry K. gibbs, and Steve Orloff. Characterization of a certified alfalfa cultivar:importance and evaluation of fall dormancy.

[3] Teuber LR, Taggard KL, Gibbs LK, McCaslin MH, Peterson MA, Barnes DK. Standard Tests to Characterize Alfalfa Cultivars Fall dormancy In: Fox C BR, Gray F, Grau C, Jessen D et al., editor. 3rd North American Alfalfa Improvement Conf: Agronomic Test; 1998. p. A-1.

[4] Kallenbach R. Estimation of Fall Dormancy in Alfalfa by Near Infrared Reflectance Spectroscopy. Crop Science. 2001;41(3):774–7.

[5] Stout DG, Hall JW. Fall growth and winter survival of alfalfa in interior British Columbia. Canadian Journal of Plant Science. 1989;69(2):491–9.

[6] Wang C, Ma BL, Han J, Wang Y, Gao Y, Hu X, et al Photoperiod Effect on Phytochrome and Abscisic Acid in Alfalfa Varieties Differing in Fall Dormancy. Journal of Plant Nutrition. 2008;31(7):1257–69. 10.1080/01904160802135027

[7] Fan WN, Yan XB, Shi YH, Wang CZ. Effects of Photoperiod and Temperature on phyA and phyB mRNA Expression of Alfalfa. Acta Agrestia Sinica. 2011;19(6):975–82.

[8] Hong-Qi DU, Liang MG, Fan WN, Peng-Ju WU, Wang CZ. Expression Levels of PHYA and PHYB mRNA in Different Fall Dormancy Alfalfa Varieties. Acta Agrestia Sinica. 2014;22(3):572–8.

[9] Tysdal HM. Influence of light, temperature, and soil moisture on the hardening process in alfalfa. J Agric Res. 1933;46:483–515.

[10] Shih SC, Jung G.A., and Shelton D.C. Effects of temperature and photoperiod on metabolic changes in alfalfa in relation to cold hardiness. Crop Sci. 1967;7:385–9.

[11] Brouwer DJ, Duke SH, Osborn TC. Mapping genetic factors associated with winter hardiness, fall growth, and freezing injury in autotetraploid alfalfa. Crop Science. 2000;40(5):1387–96.

[12] Fan W, Zhang S, Du H, Sun X, Shi Y, Wang C. Genome-Wide Identification of Different Dormant *Medicago sativa* L. MicroRNAs in Response to Fall Dormancy. PLoS ONE. 2014;9(12):e114612 10.1371/journal.pone.0114612 25473944 PMC4256440

[13] Zhang S, Shi Y, Cheng N, Du H, Fan W, Wang C. De Novo Characterization of Fall Dormant and Nondormant Alfalfa (Medicago sativa L.) Leaf Transcriptome and Identification of Candidate Genes Related to Fall Dormancy. Plos One. 2015;10(3):e0122170 10.1371/journal.pone.0122170 25799491 PMC4370819

[14] Fan WN, Sun XG, Jun-Xia NI, Hong-Qi DU, Shi YH, Yan XB, et al Effect of photoperiod on phytochromes and endogenous hormones of alfalfa with different fall-dormancies. Acta Prataculturae Sinica. 2014;23(1):177–84.

[15] Cao Y, Cao ZZ, Shi S, Tian Y. A MODIFIED TECHNIQUE MEASURING LEAF AREA OF ALFALFA. Pratacultural Science. 1990.

[16] Grabherr MG, Haas BJ, Yassour M, Levin JZ, Thompson DA, Amit I, et al Full-length transcriptome assembly from RNA-Seq data without a reference genome. Nature Biotechnology. 2011;29(7):644 10.1038/nbt.1883 21572440 PMC3571712

[17] Null RCTR, Team R, Null RCT, Core Writing T, Null R,., Team R, et al R: A language and environment for statistical computing. Computing. 2013;1:12–21.

[18] Livak KJ, Schmittgen TD. Analysis of relative gene expression data using real-time quantitative PCR and the 2(-Delta Delta C(T)) Method. Methods. 2001;25(4):402–8. 10.1006/meth.2001.1262 11846609

[19] Awai K, Maréchal E, Block MA, Brun D, Masuda T, Shimada H, et al Two types of MGDG synthase genes, found widely in both 16:3 and 18:3 plants, differentially mediate galactolipid syntheses in photosynthetic and nonphotosynthetic tissues in Arabidopsis thaliana. Proceedings of the National Academy of Sciences of the United States of America. 2001;98(19):10960–5. 10.1073/pnas.181331498 . 11553816 PMC58581

[20] Cox M, Lehninger AL, Nelson DR. Lehninger principles of biochemistry New York: Worth Publishers; 2000 p. 306–8.

[21] Kotake T, Hojo S, Yamaguchi D, Aohara T, Konishi T, Tsumuraya Y. Properties and Physiological Functions of UDP-Sugar Pyrophosphorylase in Arabidopsis. Bioscience, Biotechnology, and Biochemistry. 2007;71(3):761–71. 10.1271/bbb.60605 17341835

[22] Ahmad M, Cashmore AR. HY4 gene of A. thaliana encodes a protein with characteristics of a blue-light photoreceptor. Nature. 1993;366:162–6 10.1038/366162a0 8232555

[23] Canamero RC, Bakrim N, Bouly J-P, Garay A, Dudkin EE, Habricot Y, et al Cryptochrome photoreceptors cry1 and cry2 antagonistically regulate primary root elongation in Arabidopsis thaliana. Planta. 2006;224(5):995–1003. 10.1007/s00425-006-0280-6 16703358

[24] Tang W, Wang W, Chen D, Ji Q, Jing Y, Wang H, et al Transposase-Derived Proteins FHY3/FAR1 Interact with PHYTOCHROME-INTERACTING FACTOR1 to Regulate Chlorophyll Biosynthesis by Modulating HEMB1 during Deetiolation in Arabidopsis. The Plant Cell Online. 2012;24(5):1984–2000. 10.1105/tpc.112.097022 22634759 PMC3442582

[25] Quail P, Hudson M, Ringli C, Boylan M. the FAR1 locus encodes a novel nuclear protein specific to phytochrome a signalling. Genes and Development. 1999;13:2017–27. 10444599 10.1101/gad.13.15.2017 PMC316922

[26] Manfield IW, Devlin PF, Jen C-H, Westhead DR, Gilmartin PM. Conservation, convergence, and divergence of light-responsive, circadian-regulated, and tissue-specific expression patterns during evolution of the Arabidopsis GATA gene family. Plant physiology. 2007;143(2):941–58. 10.1104/pp.106.090761 17208962 PMC1803723

[27] Reyes JC, Muro-Pastor MI, Florencio FJ. The GATA family of transcription factors in Arabidopsis and rice. Plant physiology. 2004;134(4):1718–32. 10.1104/pp.103.037788 15084732 PMC419845

[28] Riechmann J, Heard J, Martin G, Reuber L, Keddie J, Adam L, et al Arabidopsis transcription factors: genome-wide comparative analysis among eukaryotes. Science. 2000;290(5499):2105–10. 11118137 10.1126/science.290.5499.2105

[29] Wetzel CM, Harmacek LD, Yuan LH, Wopereis JLM, Chubb R, Turini P. Loss of chloroplast protease SPPA function alters high light acclimation processes in Arabidopsis thaliana L. (Heynh.). Journal of experimental botany. 2009;60(6):1715–27. 10.1093/jxb/erp051 . 19349419 PMC2671626

[30] Lensch M, Herrmann RG, Sokolenko A. Identification and characterization of SppA, a novel light-inducible chloroplast protease complex associated with thylakoid membranes. J Biol Chem. 2001;276(36):33645–51. 10.1074/jbc.M100506200 . 11443110

[31] Hoenicka H, Nowitzki O, Hanelt D, Fladung M. Heterologous overexpression of the birch FRUITFULL-like MADS-box gene BpMADS4 prevents normal senescence and winter dormancy in Populus tremula L2008. 1001–11 p. 10.1007/s00425-007-0674-0 18185941

[32] Mérida A, Rodríguez-Galán JM, Vincent C, Romero JM. Expression of the Granule-Bound Starch Synthase I (Waxy) Gene from Snapdragon Is Developmentally and Circadian Clock Regulated. Plant Physiology. 1999;120(2):401–10. 10.1104/pp.120.2.401 10364391 PMC59278

[33] Won C, Shen X, Mashiguchi K, Zheng Z, Dai X, Cheng Y, et al Conversion of tryptophan to indole-3-acetic acid by TRYPTOPHAN AMINOTRANSFERASES OF ARABIDOPSIS and YUCCAs in Arabidopsis. Proceedings of the National Academy of Sciences. 2011;108(45):18518–23. 10.1073/pnas.1108436108 PMC3215067 22025721

[34] Bartel B, Fink GR. ILR1, an amidohydrolase that releases active indole-3-acetic acid from conjugates. Science. 1995;268(5218):1745–8. 7792599 10.1126/science.7792599

[35] Guilfoyle TJ, Hagen G. Auxin response factors. Current Opinion in Plant Biology. 2007;10(5):453–60. 10.1016/j.pbi.2007.08.014. 17900969

[36] Liscum E, Reed JW. Genetics of Aux/IAA and ARF action in plant growth and development2002. 387–400 p. 12036262

[37] Ma Y, Szostkiewicz I, Korte A, Moes D, Yang Y, Christmann A, et al Regulators of PP2C phosphatase activity function as abscisic acid sensors. Science (New York, NY). 2009;324(5930):1064–8. 10.1126/science.1172408 . 19407143

[38] Nishimura N, Sarkeshik A, Nito K, Park S-Y, Wang A, Carvalho PC, et al PYR/PYL/RCAR family members are major in-vivo ABI1 protein phosphatase 2C-interacting proteins in Arabidopsis. Plant J. 2010;61(2):290–9. 10.1111/j.1365-313X.2009.04054.x . 19874541 PMC2807913

[39] Park S-Y, Fung P, Nishimura N, Jensen DR, Fujii H, Zhao Y, et al Abscisic acid inhibits type 2C protein phosphatases via the PYR/PYL family of START proteins. Science (New York, NY). 2009;324(5930):1068–71. 10.1126/science.1173041 . 19407142 PMC2827199

[40] Saavedra X, Modrego A, Rodríguez D, González-García MP, Sanz L, Nicolás G, et al The nuclear interactor PYL8/RCAR3 of Fagus sylvatica FsPP2C1 is a positive regulator of abscisic acid signaling in seeds and stress. Plant physiology. 2010;152(1):133–50. 10.1104/pp.109.146381 . 19889877 PMC2799352

[41] Alves MS, Fontes EPB, Fietto LG. EARLY RESPONSIVE to DEHYDRATION 15, a new transcription factor that integrates stress signaling pathways. Plant Signal Behav. 2011;6(12):1993–6. 10.4161/psb.6.12.18268 22105026 PMC3337193

[42] Kariola T, Brader G, Helenius E, Li J, Heino P, Palva ET. EARLY RESPONSIVE TO DEHYDRATION 15, a Negative Regulator of Abscisic Acid Responses in Arabidopsis. Plant Physiology. 2006;142(4):1559–73. 10.1104/pp.106.086223 17056758 PMC1676049

[43] Edwards A, Heckmann AB, Yousafzai F, Duc G, Downie JA. Structural Implications of Mutations in the Pea SYM8 Symbiosis Gene, the DMI1 Ortholog, Encoding a Predicted Ion Channel. Molecular Plant-Microbe Interactions. 2007;20(10):1183–91. 10.1094/MPMI-20-10-1183 17918620

[44] Koh S, Wiles AM, Sharp JS, Naider FR, Becker JM, Stacey G. An Oligopeptide Transporter Gene Family in Arabidopsis. Plant Physiology. 2002;128(1):21–9. 10.1104/pp.010332 11788749 PMC148940

[45] Aurora Lara-Nu´n˜ez NdJsaJMVz-R. Maize D4;1 and D5 cyclin proteins in germinating maize. Associated kinase activity and regulation by phytohormones. Physiologia Plantarum. 2008;132:79–88. 10.1111/j.1399-3054.2007.00995.x 18251872

[46] Riou-Khamlichi C, Menges M, Healy JMS, Murray JAH. Sugar Control of the Plant Cell Cycle: Differential Regulation of Arabidopsis D-Type Cyclin Gene Expression. Molecular and Cellular Biology. 2000;20(13):4513–21. 10.1128/mcb.20.13.4513-4521.2000 10848578 PMC85832

[47] Barford D. Structural insights into anaphase-promoting complex function and mechanism. Philosophical Transactions of the Royal Society B: Biological Sciences. 2011;366(1584):3605–24. 10.1098/rstb.2011.0069 22084387 PMC3203452

[48] Hansen DV, Loktev AV, Ban KH, Jackson PK. Plk1 Regulates Activation of the Anaphase Promoting Complex by Phosphorylating and Triggering SCFβTrCP-dependent Destruction of the APC Inhibitor Emi1. Molecular Biology of the Cell. 2004;15(12):5623–34. 10.1091/mbc.E04-07-0598 15469984 PMC532041

[49] Jin L, Williamson A, Banerjee S, Philipp I, Rape M. Mechanism of Ubiquitin-Chain Formation by the Human Anaphase-Promoting Complex. Cell. 2008;133(4):653–65. 10.1016/j.cell.2008.04.012. 18485873 PMC2696189

[50] Hemsley P.A. TL, Grierson C.S. S-acylation: dynamic control of plant development and sigalling by lipid modification of proteins. Proceedings of the 18th international conference on Arabidopsis research; Beijing2007.

[51] Schiefelbein J, Galway M, Masucci J, Ford S. Pollen Tube and Root-Hair Tip Growth Is Disrupted in a Mutant of Arabidopsis thaliana. Plant Physiology. 1993;103(3):979–85. 10.1104/pp.103.3.979 8022944 PMC159072

[52] Ryan E, Grierson CS, Cavell A, Steer M, Dolan L. TIP1 is required for both tip growth and non-tip growth in Arabidopsis. New Phytologist. 1998;138(1):49–58. 10.1046/j.1469-8137.1998.00896.x.

[53] Sedbrook JC, Carroll KL, Hung KF, Masson PH, Somerville CR. The Arabidopsis SKU5 gene encodes an extracellular glycosyl phosphatidylinositol-anchored glycoprotein involved in directional root growth2002. 1635–48 p. 10.1105/tpc.002360 PMC150712 12119380

[54] Van Aken O, Pecenkov, aacute, T, van de Cotte B, De Rycke R, et al. Mitochondrial type-I prohibitins of Arabidopsis thaliana are required for supporting proficient meristem development2007. 850–64 p. 10.1111/j.1365-313X.2007.03276.x 17883375

[55] Zhang M, Zhang B, Qian Q, Yu Y, Li R, Zhang J, et al Brittle Culm 12, a dual-targeting kinesin-4 protein, controls cell-cycle progression and wall properties in rice. The Plant Journal. 2010;63(2):312–28. 10.1111/j.1365-313X.2010.04238.x 20444225 PMC3440585

[56] Vernos I, Raats J, Hirano T, Heasman J, Karsenti E, Wylie C. Xklp15 a chromosomal xenopus kinesin-like protein essential for spindle organization and chromosome positioning. Cell. 1995;81(1):117–27. 10.1016/0092-8674(95)90376-3. 7720067

[57] Goshima G, Vale RD. The roles of microtubule-based motor proteins in mitosis: comprehensive RNAi analysis in the Drosophila S2 cell line. The Journal of Cell Biology. 2003;162(6):1003–16. 10.1083/jcb.200303022 12975346 PMC2172859

[58] Kwon M, Morales-Mulia S, Brust-Mascher I, Rogers GC, Sharp DJ, Scholey JM. The Chromokinesin, KLP3A, Drives Mitotic Spindle Pole Separation during Prometaphase and Anaphase and Facilitates Chromatid Motility. Molecular Biology of the Cell. 2004;15(1):219–33. 10.1091/mbc.E03-07-0489 14528012 PMC307542

[59] Kurasawa Y, Earnshaw WC, Mochizuki Y, Dohmae N, Todokoro K. Essential roles of KIF4 and its binding partner PRC1 in organized central spindle midzone formation2004 2004-08-18 00:00:00. 3237–48 p. 10.1038/sj.emboj.7600347 PMC514520 15297875

[60] Mazumdar M, Sundareshan S, Misteli T. Human chromokinesin KIF4A functions in chromosome condensation and segregation. The Journal of Cell Biology. 2004;166(5):613–20. 10.1083/jcb.200401142 15326200 PMC2172419

[61] Zhu C, Jiang W. Cell cycle-dependent translocation of PRC1 on the spindle by Kif4 is essential for midzone formation and cytokinesis. Proceedings of the National Academy of Sciences of the United States of America. 2005;102(2):343–8. 10.1073/pnas.0408438102 15625105 PMC544298

[62] Harvey S, Krien M, O'Connell M. Structural maintenance of chromosomes (SMC) proteins, a family of conserved ATPases. Genome Biology. 2002;3(2):reviews3003.1—reviews.5. 10.1186/gb-2002-3-2-reviews3003 11864377 PMC139016

[63] Losada A, Hirano M, Hirano T. Identification of Xenopus SMC protein complexes required for sister chromatid cohesion. Genes & Development. 1998;12(13):1986–97. 10.1101/gad.12.13.1986 9649503 PMC316973

[64] Chen P, Zhao J, Wang Y, Wang M, Long H, Liang D, et al H3.3 actively marks enhancers and primes gene transcription via opening higher-ordered chromatin. Genes & Development. 2013;27(19):2109–24. 10.1101/gad.222174.113 24065740 PMC3850095

[65] Li Z, Reighard GL, Abbott AG, Bielenberg DG. Dormancy-associated MADS genes from the EVG locus of peach [Prunus persica (L.) Batsch] have distinct seasonal and photoperiodic expression patterns. Journal of Experimental Botany. 2009;60(12):3521–30. 10.1093/jxb/erp195 19553369 PMC2724702

[66] Chen X, Zhang Z, Liu D, Zhang K, Li A, Mao L. SQUAMOSAPromoter-Binding Protein-Like Transcription Factors: Star Players for Plant Growth and Development Journal of Integrative Plant Biology. 2010;52(11):946–51. 10.1111/j.1744-7909.2010.00987.x 20977652

[67] Klein J, Saedler H, Huijser P. A new family of DNA binding proteins includes putative transcriptional regulators of theAntirrhinum majus floral meristem identity geneSQUAMOSA. Molec Gen Genet. 1996;250(1):7–16. 10.1007/BF02191820 8569690

[68] Yoshida Y, Adachi E, Fukiya K, Iwai K, Tanaka K. Glycoprotein‐specific ubiquitin ligases recognize N‐glycans in unfolded substrates2005 2005-03-01 00:00:00. 239–44 p. 10.1038/sj.embor.7400351 PMC1299261 15723043

[69] Yoshida Y, Tokunaga F, Chiba T, Iwai K, Tanaka K, Tai T. Fbs2 Is a New Member of the E3 Ubiquitin Ligase Family That Recognizes Sugar Chains. Journal of Biological Chemistry. 2003;278(44):43877–84. 10.1074/jbc.M304157200 12939278

[70] Schuberth C, Buchberger A. UBX domain proteins: major regulators of the AAA ATPase Cdc48/p97. Cell Mol Life Sci. 2008;65(15):2360–71. 10.1007/s00018-008-8072-8 18438607 PMC11131665

[71] Stone SL, Hauksdóttir H, Troy A, Herschleb J, Kraft E, Callis J. Functional Analysis of the RING-Type Ubiquitin Ligase Family of Arabidopsis. Plant Physiology. 2005;137(1):13–30. 10.1104/pp.104.052423 15644464 PMC548835

[72] Wagner TA, Kohorn BD. Wall-Associated Kinases Are Expressed throughout Plant Development and Are Required for Cell Expansion. The Plant Cell Online. 2001;13(2):303–18. 10.1105/tpc.13.2.303 PMC102244 11226187

[73] Lally D, Ingmire P, Tong HY, He ZH. Antisense expression of a cell wall-associated protein kinase, WAK4, inhibits cell elongation and alters morphology2001. 1317–31 p. 10.1105/tpc.13.6.1317 PMC135583 11402163

[74] Pellegrini LGd, Monteiro ALG, Neumann M, Moraes de A, Pellegrin ACRSd, et al Production and quality of annual ryegrass submitted to nitrogen fertilization under grazing by lambs. Revista Brasileira de Zootecnia. 2010;39(9):1894–904.

[75] Beemster GTS, Fiorani F, Inzé D. Cell cycle: the key to plant growth control? Trends in Plant Science. 2003;8(4):154–8. 10.1016/S1360-1385(03)00046-3. 10.1016/S1360-1385(03)00046-3 12711226

[76] Williams M. Introduction to phytohormones. The Plant Cell. 2010;22:1–9. 10.1105/tpc.109.220110 20061550 PMC2828698

[77] Wolters H, Jürgens G. Survival of the flexible: hormonal growth control and adaptation in plant development. Nature Reviews Genetics. 2009;10(5):305–17. 10.1038/nrg2558 19360022

[78] Gray WM. Hormonal Regulation of Plant Growth and Development. Plos Biology. 2004;2(9):E311 10.1371/journal.pbio.0020311 15367944 PMC516799

[79] Muschietti J, McCormick S. Abscisic acid (ABA) receptors: light at the end of the tunnel2010. 10.3410/B2-15 PMC2948352 20948817

[80] Zhao Y. Auxin Biosynthesis and Its Role in Plant Development. Annual Review of Plant Biology. 2010;61(1):49–64. 10.1146/annurev-arplant-042809-112308 . 20192736 PMC3070418

[81] Staswick P. Plant hormone conjugation: A signal decision. Plant Signal Behav. 2009;4(8):757 10.1104/pp.109.138529 19820345 PMC2801392

[82] Magnus V, Hangarter RP, Good NE. Interaction of free indole-3-acetic acid and its amino acid conjugates in tomato hypocotyl cultures. Journal of Plant Growth Regulation. 1992;11(2):67–75.

[83] Sultan SE. Plant developmental responses to the environment: eco-devo insights. Current Opinion in Plant Biology. 2010;13(1):96–101. 10.1016/j.pbi.2009.09.021. 19857987

[84] Rinne P, Saarelainen A, Junttila O. Growth cessation and bud dormancy in relation to ABA level in seedlings and coppice shoots of Betula pubescens as affected by a short photoperiod, water stress and chilling. Physiologia Plantarum. 1994;90(3):451–8. 10.1111/j.1399-3054.1994.tb08801.x

[85] Millar AA, Jacobsen JV, Ross JJ, Helliwell CA, Poole AT, Scofield G, et al Seed dormancy and ABA metabolism in Arabidopsis and barley: the role of ABA 8′-hydroxylase. The Plant Journal. 2006;45(6):942–54. 10.1111/j.1365-313X.2006.02659.x 16507085

[86] Rodríguez-Gacio MdC, Matilla-Vázquez MA, Matilla AJ. Seed dormancy and ABA signaling: the breakthrough goes on. Plant Signal Behav. 2009;4(11):1035–49. 10.4161/psb.4.11.9902 . 19875942 PMC2819511

[87] Guskov A, Kern J, Gabdulkhakov A, Broser M, Zouni A, Saenger W. Cyanobacterial photosystem II at 2.9-A resolution and the role of quinones, lipids, channels and chloride. Nat Struct Mol Biol. Nature Structural & Molecular Biology. 2009;16(3):334 10.1038/nsmb.1559 19219048

[88] Umena Y, Kawakami K, Shen JR, Kamiya N. Crystal structure of oxygen-evolving photosystem II at a resolution of 1.9[thinsp]A. Nature. 2011;473(7345):55 10.1038/nature09913 21499260

[89] Wu W, Ping W, Wu H, Li M, Gu D, Xu Y. Monogalactosyldiacylglycerol deficiency in tobacco inhibits the cytochrome b6f-mediated intersystem electron transport process and affects the photostability of the photosystem II apparatus. Biochimica et biophysica acta. 2013;1827(6):709–22. 10.1016/j.bbabio.2013.02.013 23466336

[90] Shimojima M, Ohta H, Iwamatsu A, Masuda T, Shioi Y, Takamiya K. Cloning of the gene for monogalactosyldiacylglycerol synthase and its evolutionary origin. Proceedings of the National Academy of Sciences. 1997;94(1):333–7. 10.1073/pnas.94.1.333 PMC19336 8990209

[91] Dörmann Peter, Benning Christoph. Galactolipids rule in seed plants. Trends in Plant Science. 2002;7(3):112–8. 11906834 10.1016/s1360-1385(01)02216-6

[92] Jarvis P, Dörmann P, Peto CA, Lutes J, Benning C, Chory J. Galactolipid deficiency and abnormal chloroplast development in the Arabidopsis MGD synthase 1 mutant. Proceedings of the National Academy of Sciences of the United States of America. 2000;97(14):8175–9. 10.1073/pnas.100132197 10869420 PMC16689

[93] Kobayashi K, Kondo M, Fukuda H, Nishimura M, Ohta H. Galactolipid Synthesis in Chloroplast Inner Envelope Is Essential for Proper Thylakoid Biogenesis, Photosynthesis, and Embryogenesis. Proceedings of the National Academy of Sciences. 2007;104(43):17216–21. 10.1073/pnas.0704680104 PMC2040463 17940034

[94] Stitt M, Schulze D. Does Rubisco control the rate of photosynthesis and plant growth? An exercise in molecular ecophysiology. Plant, Cell & Environment. 1994;17(5):465–87. 10.1111/j.1365-3040.1994.tb00144.x

[95] Portis R. Activation ofRibulose-1,5Bis phosphate Carboxylase/ Oxygenase (Rubisco) byRubisco Activasel Effects ofSomeSugarPhosphates. 1990;94(1):245–50.

[96] Feller U, Craftsbrandner SJ, Salvucci ME. Moderately High Temperatures Inhibit Ribulose-1,5-Bisphosphate Carboxylase/Oxygenase (Rubisco) Activase-Mediated Activation of Rubisco. Plant Physiology. 1998;116(2):539–46. 9490757 10.1104/pp.116.2.539 PMC35111

[97] Hudson GS, Caemmerer SV, Evans JR, Andrews TJ. Reduction of Ribulose Bisphosphate Carboxylase Activase Levels in Tobacco (Nicotiana tabacum) by Antisense RNA Reduces Ribulose Bisphosphate Carboxylase Carbamylation and Impairs Photosynthesis. Plant Physiology. 1993;102(4):1119–28. 8278543 10.1104/pp.102.4.1119 PMC158896

[98] Mate CJ, Caemmerer SV, Evans JR, Hudson GS, Andrews TJ. The drelationship between CO 2 -assimilation rate, Rubisco carbamylation and Rubisco activase content in activase-deficient transgenic tobacco suggests a simple model of activase action. Planta. 1996;198(4):604–13. 10.1007/BF00262648 28321671

[99] Jiang CZ, Paul QW, Rhu A, Daniel K, Rodermel SR. Antisense RNA inhibition of Rubisco activase expression. Plant Journal. 2010;5(6):787–98.

[100] Kurek I, Chang TK, Bertain SM, Madrigal A, Liu L, Lassner MW, et al Enhanced Thermostability of Arabidopsis Rubisco Activase Improves Photosynthesis and Growth Rates under Moderate Heat Stress. Plant Cell. 2007;19(10):3230 10.1105/tpc.107.054171 17933901 PMC2174701

[101] Kumar A, Li C, Jr ARP. Arabidopsis thaliana expressing a thermostable chimeric Rubisco activase exhibits enhanced growth and higher rates of photosynthesis at moderately high temperatures. Photosynthesis Research. 2009;100(3):143–53. 10.1007/s11120-009-9438-y 19507049

