# Supplementary material for: Screening and identification of key genes regulating fall dormancy in alfalfa leaves
Source: PLoS One. 2017 Dec 6;12(12):e0188964. doi: 10.1371/journal.pone.0188964 (PMC5718555; doi:10.1371/journal.pone.0188964)
Supplement: S1 Table — (DOCX) [file pone.0188964.s001.docx]

Table S1 QRT-PCR primers of 37 differentially expressed genes

| genes | primer | primer sequence |
| --- | --- | --- |
| Probable mediator of RNA polymerase II | comp53272s | TTTTATGAACGAGTTGAAGAG |
|  | comp53272a | TCAGAGTGGTAAGAAAGTGTC |
| Monocopper oxidase-like protein SKU5 | comp32326s | GGAACACCAAGAGGAGAAGCA |
|  | comp32326a | TTTTCATCCTCAACATTGCTCTG |
| Putative E3 ubiquitin-protein ligase LIN | comp909276s | GCATATATAAGATAGCAAGGGG |
|  | comp909276a | AAAGTGTAATAAGAGGACAGCG |
| Monosaccharide-sensing protein 3 | comp70285s | ACTGACAGCTGAAATTGCGGCA |
|  | comp70285a | AGCAGGCAGGAGTAGGGGC |
| Protein S-acyltransferase 24 (PAT24) | comp686013s | CGGTTGTAGTTGTTAGCGTTGG |
|  | comp686013a | TTGGAGGCTTGCAGGGTATT |
| BTB/POZ domain-containing protein At1g67900 | comp538610s | ATGTTCTAAGTTAAATAATGTGCTA |
|  | comp538610a | GTTTTTCTTTCCCTCTTGTCA |
| BTB/POZ domain-containing protein At3g19850 | comp933338s | AGAGGTAAAAGAAATGTTGAGGT |
|  | comp933338a | TGAATATGCAGATTGCTATGGTC |
| UBX domain-containing protein 7 | comp655910s | GAGCCACTAAACATCGGATAAAGA |
|  | comp655910a | GCTGATGCTTCGATTAAGTCTTCT |
| Protein early responsive to dehydration 15 | comp61323s | TAACAATCATAACATCACTCTGC |
|  | comp61323a | AAAAATCCTCCACTTGACGAAAT |
| Methylesterase17 | comp343499s | GCAAAAGAAGAGAGGGAGCATC |
|  | comp343499a | AGCACCAACCTCCTCCACCTAT |
| Squamosa promoter-binding protein 1(SBP1) | comp50413s | ACGGAATGAGCCTTAGAATGGT |
|  | comp50413a | TTGGGGTGAGGGAAAGAGAAT |
| Structural maintenance of chromosomes protein 3(SMC3 | comp37666s | GTTGAAATGCGTGGCTGTGATAC |
|  | comp37666a | CGGTAAGTTCTTTCTCCCTGCTC |
| ABC transporter B family member 11 | comp10285s | TTTGATGGAGAAAAACACAAGAGG |
|  | comp10285a | CTAGTGCATCCCCAACCAGAGC |
| Monogalactosyldiacylglycerol synthase 3 | comp1260s | CATACAATTTGGAACCGTTGG |
|  | comp1260a | TAAGAGGCTGACCTTGCTGGA |
| Cryptochrome-2(CRY-2) | comp46970s | CTGCTTCAGCCACCACCTC |
|  | comp46970a | ATGAAAAATCAGAAAATGGGTATG |
| Abscisic acid receptor PYL8 | comp57456s | ACAATGAAGAGCATACCAAGAAAACA |
|  | comp57456a | GCTTAGGAGATGGACTGGCGAT |
| F-box only protein 6 | comp29926s | CCTGTGCGAACAATCTGAAGC |
|  | comp29926a | ATCTTTCGGCAATGCGGGTAA |
| Probable histone H2A.2 | comp424089s | GGTTCTTGAGTTGGCTGGAAATG |
|  | comp424089a | GAAGCACAGGGTTAATGTTGGGA |
| IAA-amino acid hydrolase ILR1-like 1 | comp522282s | TATCCCTTAGGTGTTGTTGCGT |
|  | comp522282a | CTCCCTTGAAATAATGCTTTGC |
| Auxin response factor 6(ARF6) | comp15714s | CAACTTGTGTGGATGAATCTGGC |
|  | comp15714a | GTGACCTCTCAATTCTCAAAGGA |
| ABC(ATP-binding cassette) transporter C family member 7 | comp46282s | TTAGCCCATAAAGACCTTCCATT |
|  | comp46282a | AACATTACCACACTTCAACTCAA |
| Cyclin-D5-2 | comp33056s | ACAGAAATAGACCAAGAGGGAGA |
|  | comp33056a | CAGGGCAGAACAAGTAAACAAAG |
| Ribulose bisphosphate carboxylase/oxygenase (rubisco) activase | comp53413s | AAAACTGTATCAACCAAACCCTTCC |
|  | comp53413a | GTTGCTGCTGAGATTGATGAAAG |
| Beta-D –glucosidase | comp59094s | CATCTACTGCCTGTGAGACGAAC |
|  | comp59094a | GAACGGTATCCAAGTGATACGAG |
| probable ion channel SYM8 | comp40098s | CAAGTTCACCAGCAGCAAGTTT |
|  | comp40098a | AGAGGTACGCAAGGGCTATTTC |
| Prohibitin-3, mitochondrial | comp672512s | CGTCGGCGAAGGAACTCAT |
|  | comp672512a | TCTGTTAGCGGAGACGGATGA |
| UDP-sugar pyrophosphorylase | comp25768s | ACCAACTCTTGCTGTTTCTGC |
|  | comp25768a | CCTACCTACGTTGTTGTCTTGTCT |
| Putative hospholipid-transporting ATPase 7 | comp343967s | CAGTGATTGTCCGTGATGA |
|  | comp343967a | AAATACGCTTTTCCGTTC |
| MADS-box transcription factor PHERES 1 | comp391402s | TTCCTTGGCTTCAGTAATCT |
|  | comp391402a | GAAACTTGCCTTTATCTCCA |
| Serine/threonine-protein kinase Nek1 | comp403595s | TTGTCTAAGGTTTTGTCA |
|  | comp403595a | TCTTGAGATTAGAAGCTAA |
| E3 ubiquitin-protein ligase UPL6 | comp395328s | TGGTAATCTGTCCCGAGCAT |
|  | comp395328a | CACCTGGCAGCTTGGATG |
| Protein HEADING DATE 3A | comp46405s | TGACGCAATAATACAAACACG |
|  | comp46405a | CCAGATGCTCCTAGTCCAAG |
| WAK3 | comp41596s | AAATCCTTATGAACCCAATG |
|  | comp41596a | AAATCCTTATGAACCCAATG |
| Probable indole-3-pyruvate monooxygenase YUCCA3 | comp36708s | CCACAGCCAACAACAAGA |
|  | comp36708a | ATGGAGGATTAAGAGCAATAGG |
| Histone H3.3 | comp57595 s | CGAGTTACTATTATGCCCAAGGAC |
|  | comp57595 a | GCCACCCATCTAAACAGCACCAAA |
| Granule-bound starch synthase 1(GBSS1 | comp56985s | TCCAGGAATCAACTTTAGCG |
|  | comp56985a | ATCTCCTCGTCCCGCACA |
| Kinesin-4 | comp887354s | GGCTGAATCCAAGACTGC |
|  | comp887354a | TTAATGGTTGAGTGTCCCTAT |
