# Supplementary material for: Screening and identification of key genes regulating fall dormancy in alfalfa leaves
Source: PLoS One. 2017 Dec 6;12(12):e0188964. doi: 10.1371/journal.pone.0188964 (PMC5718555; doi:10.1371/journal.pone.0188964)
Supplement: S7 File — (DOCX) [file pone.0188964.s008.docx]

| Pathway and pathway ID | DEGs involved in pathway |
| --- | --- |
| Metabolic pathways ko01100” Starch and sucrose metabolism ko00500, Biosynthesis of secondary metabolites ko01110 “Amino sugar and nucleotide sugar metabolism ko00520, Cyanoamino acid metabolism ko00460, Phenylpropanoid biosynthesis ko00940”” | sp\|Q5W915\|USP_PEA UDP-sugar pyrophosphorylase |
|  | sp\|Q9LW07\|PGLR3_ARATH Probable polygalacturonase At3g15720 |
|  | sp\|P50668\|COX1_CHOBI Cytochrome c oxidase subunit 1 |
|  | sp\|O80689\|BGL45_ARATH Beta-glucosidase 45 |
|  | sp\|O80690\|BGL46_ARATH Beta-glucosidase 46 |
|  | sp\|Q7XSK0\|BGL18_ORYSJ Beta-glucosidase 18 |
|  | sp\|Q9SVS1\|BGL47_ARATH Beta-glucosidase 47 |
|  | sp\|Q96522\|PER45_ARATH Peroxidase 45 |
|  | sp\|Q84P21\|4CLL5_ARATH 4-coumarate--CoA ligase-like 5; sp\|Q8RU95\|4CLL6_ORYSJ 4-coumarate--CoA ligase-like 6 |
|  | sp\|Q37620\|COX3_PROWI Cytochrome c oxidase subunit 3 |
|  | sp\|F2Y4A3\|SPHK2_ARATH Sphingosine kinase 2 |
|  | sp\|Q9SI93\|MGDG3_ARATH Monogalactosyldiacylglycerol synthase 3, chloroplastic |
|  | sp\|Q8VYH2\|ERG16_ARATH Squalene epoxidase 3 |
|  | sp\|P17859\|AMYA_VIGMU Alpha-amylase |
| Tryptophan metabolism ko00380 | sp\|O23024\|YUC3_ARATH Probable indole-3-pyruvate monooxygenase YUCCA3 |
| RNA transport ko03013 | sp\|P55852\|SUMO1_ARATH Small ubiquitin-related modifier 1 |
|  | sp\|P84316\|EF1A_HELZE Elongation factor 1-alpha (Fragment); sp\|P34825\|EF1A_HYPJE Elongation factor 1-alpha |
|  | sp\|P38548\|RAN_VICFA GTP-binding nuclear protein Ran/TC4; sp\|P38546\|RAN1_SOLLC GTP-binding nuclear protein Ran1; sp\|Q8H156\|RAN3_ARATH GTP-binding nuclear protein Ran-3 |
|  | sp\|Q8VDW0\|DX39A_MOUSE ATP-dependent RNA helicase DDX39A |
|  | sp\|O59949\|EF1A_YARLI Elongation factor 1-alpha |
| Ubiquitin mediated proteolysis ko04120 | sp\|Q9FFF9\|APC1_ARATH Anaphase-promoting complex subunit 1 |
|  | sp\|Q8RWB8\|UPL6_ARATH E3 ubiquitin-protein ligase UPL6 |
| Ribosome ko03010 | sp\|P27075\|RL44_CANTR 60S ribosomal protein L44; sp\|P27074\|RL44Q_CANMA 60S ribosomal protein L44 Q |
|  | sp\|Q9SGA6\|RS191_ARATH 40S ribosomal protein S19-1 |
|  | sp\|O16797\|RL3_DROME 60S ribosomal protein L3 |
|  | sp\|P62282\|RS11_RAT 40S ribosomal protein S11 |
|  | sp\|Q4GXG7\|RL18_TIMBA 60S ribosomal protein L18 |
|  | sp\|P48159\|RL23_DROME 60S ribosomal protein L23 |
|  | sp\|Q6XIM8\|RS15A_DROYA 40S ribosomal protein S15a |
|  | sp\|Q5UAP4\|RSSA_BOMMO 40S ribosomal protein SA |
|  | sp\|O76756\|RS8_APIME 40S ribosomal protein S8 |
|  | sp\|Q33BZ6\|RK14_NICTO 50S ribosomal protein L14, chloroplastic |
|  | sp\|Q7RVN0\|RL11_NEUCR 60S ribosomal protein L11 |
|  | sp\|Q7RVI1\|RS5_NEUCR 40S ribosomal protein S5 |
|  | sp\|Q54UG4\|RL37A_DICDI 60S ribosomal protein L37a |
| Pentose and glucuronate interconversions ko00040 | sp\|Q9LW07\|PGLR3_ARATH Probable polygalacturonase At3g15720 |
|  | sp\|Q5W915\|USP_PEA UDP-sugar pyrophosphorylase |
| Protein processing in endoplasmic reticulum ko04141 | sp\|P36182\|HSP82_TOBAC Heat shock protein 82 (Fragment) |
|  | sp\|P27322\|HSP72_SOLLC Heat shock cognate 70 kDa protein 2 |
|  | sp\|Q9LHA8\|MD37C_ARATH Probable mediator of RNA polymerase II transcription subunit 37c |
|  | sp\|Q9C5S2\|IRE1A_ARATH Serine/threonine-protein kinase/endoribonuclease IRE1a |
|  | sp\|P04793\|HSP13_SOYBN 17.5 kDa class I heat shock protein |
| Oxidative phosphorylation ko00190 | sp\|P50668\|COX1_CHOBI Cytochrome c oxidase subunit 1 |
|  | sp\|Q03194\|PMA4_NICPL Plasma membrane ATPase 4 |
|  | sp\|Q37620\|COX3_PROWI Cytochrome c oxidase subunit 3 |
| Endocytosis ko04144 | sp\|P27322\|HSP72_SOLLC Heat shock cognate 70 kDa protein 2 |
|  | sp\|Q9U639\|HSP7D_MANSE Heat shock 70 kDa protein cognate 4 |
|  | sp\|Q9LHA8\|MD37C_ARATH Probable mediator of RNA polymerase II transcription subunit 37c |
| Plant-pathogen interaction ko04626 | sp\|P36182\|HSP82_TOBAC Heat shock protein 82 (Fragment) |
|  | sp\|Q42396\|CDPKC_ARATH Calcium-dependent protein kinase 12 |
| Spliceosome ko03040 | sp\|P27322\|HSP72_SOLLC Heat shock cognate 70 kDa protein 2 |
|  | sp\|Q9U639\|HSP7D_MANSE Heat shock 70 kDa protein cognate 4 |
|  | sp\|Q8VDW0\|DX39A_MOUSE ATP-dependent RNA helicase DDX39A |
|  | sp\|Q9LHA8\|MD37C_ARATH Probable mediator of RNA polymerase II transcription subunit 37c |
| Ribosome biogenesis in eukaryotes ko03008 | sp\|P38548\|RAN_VICFA GTP-binding nuclear protein Ran/TC4; sp\|P38546\|RAN1_SOLLC GTP-binding nuclear protein Ran1; sp\|Q8H156\|RAN3_ARATH GTP-binding nuclear protein Ran-3 |
| Base excision repair ko03410 | sp\|Q9MB73\|LGT_CITUN Limonoid UDP-glucosyltransferase; sp\|P40620\|HMGL_VICFA HMG1/2-like protein |
|  | sp\|Q24537\|HMG2_DROME High mobility group protein DSP1 |
| alpha-Linolenic acid metabolism ko00592 | sp\|Q84P21\|4CLL5_ARATH 4-coumarate--CoA ligase-like 5; sp\|Q8RU95\|4CLL6_ORYSJ 4-coumarate--CoA ligase-like 6 |
| Sphingolipid metabolism ko00600 | sp\|F2Y4A3\|SPHK2_ARATH Sphingosine kinase 2 |
| Circadian rhythm – plant ko04712 | sp\|Q96524\|CRY2_ARATH Cryptochrome-2 |
| Phenylalanine metabolism ko00360, Phenylpropanoid biosynthesis ko00940 | sp\|Q96522\|PER45_ARATH Peroxidase 45 |
| mRNA surveillance pathway ko03015 | sp\|Q8VDW0\|DX39A_MOUSE ATP-dependent RNA helicase DDX39A |
| Plant hormone signal transduction ko04075 | sp\|Q9FGM1\|PYL8_ARATH Abscisic acid receptor PYL8 |
| Sesquiterpenoid biosynthesis ko00909 | sp\|Q8VYH2\|ERG16_ARATH Squalene epoxidase 3 |
| Mismatch repair ko03430 | sp\|Q9FHJ6\|RFA1C_ARATH Replication protein A 70 kDa DNA-binding subunit C |
| Glycerolipid metabolism ko00561 | sp\|Q9SI93\|MGDG3_ARATH Monogalactosyldiacylglycerol synthase 3, chloroplastic |
| DNA replication ko03030, Homologous recombination ko03440, Nucleotide excision repair ko03420 | sp\|Q9FHJ6\|RFA1C_ARATH Replication protein A 70 kDa DNA-binding subunit C |
| Glutathione metabolism ko00480 | sp\|P32110\|GSTX6_SOYBN Probable glutathione S-transferase |
| RNA degradation ko03018, Ribosome biogenesis in eukaryotes ko03008 | sp\|P92555\|M1250_ARATH Uncharacterized mitochondrial protein AtMg01250 |
